# The distorting effects of producer strategies: Why engagement does not reveal consumer preferences for misinformation

**DOI:** 10.1073/pnas.2315195121

**Published:** 2024-02-27

**Authors:** Alexander J. Stewart, Antonio A. Arechar, David G. Rand, Joshua B. Plotkin

**Affiliations:** ^a^School of Mathematics and Statistics, University of St Andrews, St Andrews KY16 9SS, United Kingdom; ^b^División de Economía, Center for Research and Teaching in Economics, Centro de Investigación y Docencia Económicas, Aguascalientes, MX 20314; ^c^Sloan School of Management, Massachusetts Institute of Technology, Cambridge, MA 02139; ^d^Department of Biology, University of Pennsylvania, Philadelphia, PA 19104

**Keywords:** misinformation, game theory, information ecosystems, online behavior

## Abstract

A great deal of empirical research has examined who falls for misinformation and why. Here, we introduce a formal game-theoretic model of engagement with news stories that captures the strategic interplay between (mis)information consumers and producers. A key insight from the model is that observed patterns of engagement do not necessarily reflect the preferences of consumers. This is because producers seeking to promote misinformation can use strategies that lead moderately inattentive readers to engage more with false stories than true ones—even when readers prefer more accurate over less accurate information. We then empirically test people’s preferences for accuracy in the news. In three studies, we find that people strongly prefer to click and share news they perceive as more accurate—both in a general population sample, and in a sample of users recruited through Twitter who had actually shared links to misinformation sites online. Despite this preference for accurate news—and consistent with the predictions of our model—we find markedly different engagement patterns for articles from misinformation versus mainstream news sites. Using 1,000 headlines from 20 misinformation and 20 mainstream news sites, we compare Facebook engagement data with 20,000 accuracy ratings collected in a survey experiment. Engagement with a headline is negatively correlated with perceived accuracy for misinformation sites, but positively correlated with perceived accuracy for mainstream sites. Taken together, these theoretical and empirical results suggest that consumer preferences cannot be straightforwardly inferred from empirical patterns of engagement.

## Significance

Online misinformation shapes public discourse and world affairs. But we have a poor understanding of the principles that govern its spread. We use game theory to study engagement with misinformation, modeling the interplay between news producers and consumers. We show that even truth-seeking consumers can be induced to engage with false stories by strategic news producers who wish to spread misinformation. We then use experiments to determine whether people prefer true or fake news. We find that consumers who engage with misinformation sites actually prefer to engage with accurate information, even while inaccurate articles from those sites generate greater overall engagement. Taken together, these results show that the way consumers engage with misinformation may not reflect their actual preferences.

False or misleading information is a fundamental problem for people trying to form an accurate understanding of the world (1). There has been widespread concern in recent years about the spread of misinformation—for example, during Brexit and the 2016 US Presidential Election (1), the COVID-19 pandemic (2, 3), and the 2020 US Presidential election (4)—among policy makers, scientists, and the general public, leading to an explosion of academic research on this topic.

Most of this prior work has been empirical in nature, using observational data and experiments to ask questions such as the following: Who reads, shares, and believes misinformation (5–10)? Why do people fall for misinformation (11–14)? What interventions can combat misinformation (15–18)? In contrast to this large body of empirical research, however, there has been very little work using formal models to explore the spread of misinformation. To this end, here we develop a game-theoretic model for the evolution of news engagement. Considering this question from a game-theoretic perspective highlights the necessity of not simply modeling consumers as isolated agents, but rather of considering the dynamics of news engagement and dissemination. Inferences about the revealed preferences of consumers that fail to account for these dynamics are likely to be misleading (19–21). To fully understand the spread of misinformation, therefore, we must examine the strategic considerations that arise on both sides of the news market (22–24).

To this end, we introduce an asymmetric “misinformation game” played between news outlets, who can choose to publish true or false information, and news consumers, who can choose whether or not to engage with stories from a given outlet. We focus on the dissemination strategies of outlets that try to generate engagement with either true or false stories, and the news engagement strategies of readers who seek to consume either true or false stories, but operate under cognitive constraints.

We show that simple strategies, which vary the rate of production of true and false stories over time, when adopted by publishers seeking to spread misinformation, can successfully induce a substantial level of engagement with false stories among truth-seeking readers who try to maximize their consumption of accurate information. In particular, our model predicts that publishers who seek to spread misinformation should adopt strategies that i) include a mix of true and false stories over time and ii) increase the rate of disseminating misinformation as the level of engagement they receive from readers increases.

Our model predicts that, as a result of strategic dissemination, outlets seeking to spread misinformation can induce somewhat inattentive readers to have greater engagement with false stories than with true stories, creating an apparent preference for misinformation among their readers; whereas outlets seeking to spread accurate information should produce the opposite pattern. Importantly, we predict that this trend will hold even when all readers in fact value accuracy, and false headlines are no more “attractive” (e.g., due to novelty, emotional appeal, etc) than accurate headlines.

We then complement these theoretical results by collecting empirical data. First, we measured the expressed preferences for engaging with accurate versus inaccurate information among 511 subjects sampled from the general population, as well as 119 Twitter users who had shared links to fake news. Both groups express a strong preference for sharing and engaging with accurate news stories. However, in a second empirical study integrating 20,000 accuracy ratings for 1,000 news stories published across 40 outlets with Facebook engagement ratings for each story from Crowdtangle (25), we also find that—in spite of the preference for accuracy demonstrated in the first studies—misinformation sites tend to generate higher engagement with less accurate stories, while mainstream sites generate the opposite pattern. Taken together, our results call into question the idea that patterns of engagement with misinformation reflect the preferences of news consumers. To properly account for the spread of misinformation, we must account for the strategies of those who produce the news, rather than just the preferences of readers.

## The Misinformation Game.

**Fig. 1.**
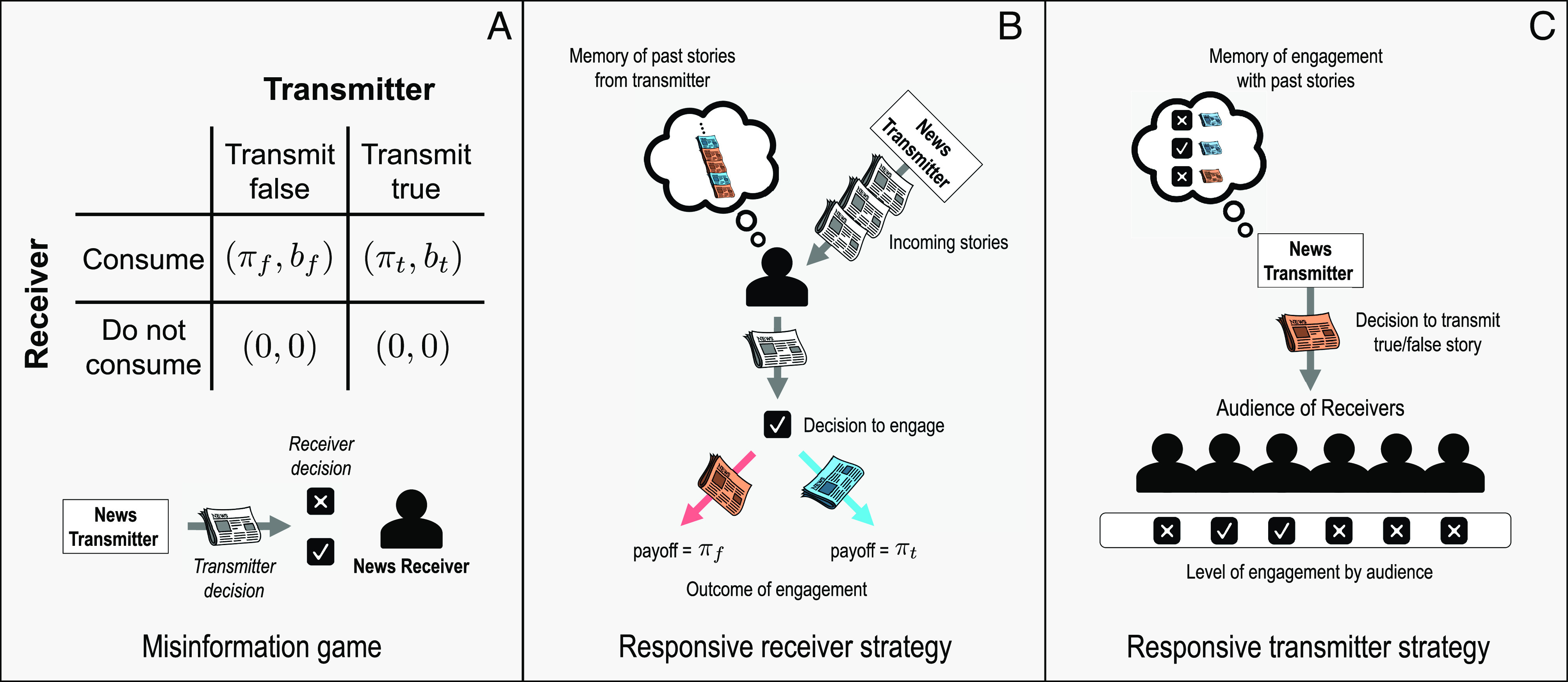
The misinformation game. (*A*) We developed the “misinformation game” in which transmitters choose whether to share true or false stories and receivers decide whether to engage with each story or not. We assume that receivers gain utility $\pi_{t}$ from engaging with accurate information and utility $\pi_{f}$ for engaging with misinformation. Receivers are incentivized to seek true news stories and avoid misinformation stories when $\pi_{t}>0$ and $\pi_{f}<0$. (*B*) We assume that, when deciding whether to engage with a given story, receivers can take into account its perceived accuracy, and the accuracy of past stories shared by transmitters. Receivers update their strategy using a myopic, noisy optimization process. (*C*) We assume that a transmitter chooses each subsequent story to be true or false based on the veracity of their previous story and the level of engagement it received. The number of independent receivers whose engagement a transmitter considers determines how precisely the transmitter can target its stories.

We develop a theory of the production and consumption of misinformation using a game-theoretic approach. We consider an asymmetric, asynchronous, infinitely repeated coordination game, which we dub the “misinformation game,” in which a news transmitter (i.e., a news outlet or a platform promoting news stories) chooses whether to transmit pieces of true or false information (i.e., news stories) while a receiver (i.e., news consumer) chooses whether or not to engage with each piece of transmitted news (Fig. 1).

Receivers derive a utility $\pi_{t}$ from engaging with true news stories, and utility $\pi_{f}$ from engaging with false stories (Fig. 1); they derive no utility if they do not engage. A payoff structure $\pi_{t}>0$ and $\pi_{f}<0$ reflects the preferences of readers who prefer accurate information and seek to avoid misinformation; a payoff structure $\pi_{t}<0$ and $\pi_{f}>0$ reflects receivers who prefer misinformation and avoid accurate information. In *SI Appendix*, sections 1 and 3.9) we also provide results for receivers who are indifferent to the veracity of news stories, i.e., for a payoff structure $\pi_{t}>0$ and $\pi_{f}>0$.

Similarly, transmitters derive benefit $b_{t}$ or $b_{f}$ from engagement by a receiver with a true or false story, and no payoff if the receiver does not engage. The differences in these benefits may reflect differences in the ease of producing such content, or some underlying ideological preference of the transmitter. We consider two types of news transmitters: a misinformation transmitter that seeks to promote misinformation stories, so that $b_{t}\;=\;0$ and $b_{f}\;>\;0$, and a mainstream transmitter that seeks to promote accurate stories, so that $b_{t}>0$ and $b_{f}=0$. In *SI Appendix*, section 3.2, we also consider a “clickbait” transmitter who simply aims to maximize engagement regardless of accuracy ($b_{t}\;=\;b_{f}\;>\;0$).

## Transmitter Strategies.

We model the dynamics of news dissemination and engagement as an optimization process, first specifying the strategy spaces available to transmitters and receivers. A transmitter can choose to share either a true or false story with a probability that depends on the level of engagement their previous story generated. We first introduce a transmitter strategy space, in which a given transmitter strategy has a baseline accuracy, along with a feedback term described by

[1] $$\begin{array}{c} r_{kt}=\alpha +\sum_{l}\gamma_{l}{\left(\frac{k}{N}\right)}^{l}\\ r_{kf}=\underset{\text{baseline accuracy}}{\underset{︸}{\beta }}+\sum_{l}\underset{\text{feedback}}{\underset{︸}{\theta_{l}{\left(\frac{k}{N}\right)}^{l}}}. \end{array}$$

Here, $r_{\mathrm{ki}}$ defines the probability of sharing a true story given that the previous story was true or false (i∈{t,f}) and that k out of N targeted receivers chose to engage with the story. The parameters $\gamma_{l}$ and $\theta_{l}$ describe the shape of the (polynomial) feedback function, where l indexes the lth order polynomial term ${\left(\frac{k}{N}\right)}^{l}$. When there is no feedback ($\gamma_{l}=\theta_{l}=0$ for all l) transmitters do not take account of past engagement. For convenience, we define $\gamma =\sum_{l}\gamma_{l}$ and $\theta =\sum_{l}\theta_{l}$. Note that in any given round of the iterated game, a transmitter must either share a true or false story, and so the overall rate of news production is constant.

## Receiver Strategies.

We assume that receivers can pay different levels, and different types, of attention when deciding whether to engage with a news story. They can attend to the likely veracity of the headline, and to their recent experience of engagement with the source that transmitted the story. In particular, a receiver engages with a story with probability $q_{\mathrm{ij}}^{m}$ where

[2] $$\begin{array}{cc} q_{\mathrm{ij}}^{m}= & \;\underset{\;\text{P}(\;\text{engage}|\;\text{true}\;\&\;\;\text{attentive})}{\underbrace{a_{0}\delta_{\mathrm{mt}}}}+\underset{\;\text{P}(\;\text{engage}|\;\text{no memory}\;\&\;\;\text{inattentive})}{\underbrace{\left(1-a_{0}\right)\left(1-a_{1}\right)p_{0}}}\\ & \;+\underset{\;\text{P}(\;\text{engage}|\;\text{memory}\;\&\;\;\text{inattentive}).}{\underbrace{\left(1-a_{0}\right)a_{1}p_{\mathrm{ij}}}} \end{array}$$

Indices i and j describe the outcome of the previous news story shared by the transmitter: index j∈{t,f} indicates whether the transmitter’s previous story was true or false and index i∈{c,n} indicates whether the receiver either engaged with (c) or did not engage with (n) the previous story. The index m∈{t,f} indicates whether the transmitter’s current story is true or false. With probability $a_{0}$ the receiver is able to directly assess the veracity of the current story (14), where $\delta_{\mathrm{mt}}$ is the Kronecker delta, equal to 1 if the newly shared story is true, and 0 otherwise. If the receiver is unable to assess the veracity of the current story (e.g., due to lack of background knowledge, or failure to pay attention), then with probability $a_{1}$ they make their decision based on their previous experience of the news source, according to a behavioral strategy $p=\{p_{\mathrm{ct}},p_{\mathrm{cf}},p_{\mathrm{nt}},p_{\mathrm{nf}}\}$, where $p_{\mathrm{ct}}$ is the probability of engaging given that they chose to engage in the previous round and the story was true, and so on. Finally, if the reader attends to neither veracity nor past experience, they engage with the story with fixed probability $p_{0}$.

## Dynamics of Reader Engagement.

The dynamics of transmission and engagement in our model occur across two different timescales. First, on short timescales, news is produced and consumed (or not) by transmitters and receivers using fixed strategies for a repeated game. Second, over longer timescales, transmitters and receivers update their strategies via a noisy optimization process (see *SI Appendix*, section 1 for full details). Thus, strategy optimization occurs with respect to the utility derived from the equilibrium payoffs across rounds of news production/consumption generated under the (infinitely) repeated misinformation game.

To understand the dynamics of reader engagement under this model, we begin by analyzing the dynamics of the repeated misinformation game for a pair of fixed transmitter and receiver strategies (*SI Appendix*, section 1, where we also give an equivalent expression for the case of multiple receivers). We show that a transmitter using a strategy of the type described by Eq. **1** enforces a specific relationship between the proportion of transmitted stories that are false, $v_{f}$, and the overall probability the receiver engages with true and false stories, $v_{\mathrm{tc}}$ and $v_{\mathrm{fc}}$:

[3] $$\begin{array}{c} v_{f}=\frac{1-\alpha }{1-\alpha +\beta }-\frac{\theta }{1-\alpha +\beta }v_{\mathrm{fc}}-\frac{\gamma }{1-\alpha +\beta }v_{\mathrm{tc}}. \end{array}$$

Remarkably, this relationship between the misinformation transmission probability per story $v_{f}$ and the receiver’s overall engagement with accurate stories, $v_{\mathrm{tc}}$ and misinformation $v_{\mathrm{fc}}$ holds regardless of the receiver’s strategy for engagement—and therefore regardless of the receiver’s preference for consuming true versus false stories. This kind of unilaterally enforced constraint, imposed by one player in a repeated game, has been extensively studied in the context of so-called “extortion strategies” in the iterated prisoner’s dilemma (26). Here, we adapt this idea for asymmetric games related to news production and consumption.

Eq. **3** holds regardless of the strategy of the receiver—and so it can be understood as a constraint enforced by the (fixed) transmitter strategy on the optimization process of any receiver. That is, Eq. **3** shows that a news site can unilaterally shape the dynamics of consumer engagement by constraining how engagement and misinformation transmission covary, as the consumer updates his strategy. For example, a transmitter can choose their strategy to ensure that the probability of production of false stores ($v_{f}$) is positively correlated with consumer engagement ($v_{\mathrm{tc}}$ and $v_{\mathrm{fc}}$, Eq. **3**), regardless of what strategies the consumer explores. When a transmitter uses this strategy, consumers who increase their overall engagement with a news source for whatever reason will always encounter greater levels of misinformation as they do so.

**Fig. 2.**
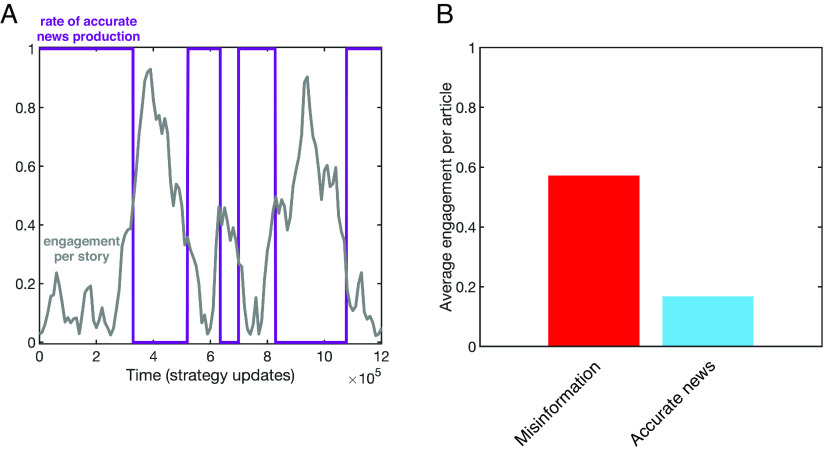
A transmitter strategy can unilaterally drive engagement with misinformation. An illustrative example of a transmitter strategy that drives engagement with misinformation. We selected a transmitter strategy that employs nonlinear feedback, in the form of a sigmoidal function $r_{\mathrm{kt}}=1/\left(1+\exp\left[\lambda \left(k/N-0.5\right)\right]\right)$ and $r_{\mathrm{kf}}=1/\left(1+\exp\left[\lambda \left(k/N-0.25\right)\right]\right)$, where we have set λ=100 and the population of receivers to be N=100 (*SI Appendix*, section 1.6). (*A*) Dynamics of production of accurate news (purple) and engagement (gray) for a receiver using a noisy optimization process with low attention to accuracy ($a_{0}=0$), experience ($a_{1}=0$) and to payoff (σ=1). The transmitter strategy produces a pattern of low levels of misinformation when engagement is low, switching to high levels of misinformation production when engagement is high. (*B*) As a result of these dynamics, the average engagement probability per article is higher for misinformation (red) than for accurate news stories (blue), even though the consumer was seeking to increase engagement with true stories.

This type of transmitter strategy is particularly important when we consider receivers who seek to increase their engagement with true stories, interacting with a transmitter who seeks to spread misinformation. When such receivers engage more with such a transmitter, they will receive increasing amounts of misinformation. If, in response, they then decrease their engagement with the transmitter’s stories they will be targeted with more true stories, and thus they are incentivized to increase their engagement once again. The result of this dynamic is a negative correlation between story accuracy and receiver engagement, even though the receiver is seeking to increase their engagement with accurate information. An illustrative example of this type of dynamic is shown in Fig. 2 and in *SI Appendix*, Fig. S2.

Critically, these results mean that the empirical observation of false stories receiving more engagement than true stories, e.g., Vosoughi et al. (27) does not necessarily imply that false stories are actually more attractive to readers—since this same pattern can be generated by a clever transmitter responding to truth-seeking consumer behavior, or may emerge spontaneously among producers engaging in their own process of “myopic” optimization, as seen in *SI Appendix*, Fig. S5. (Whereas in the absence of any feedback between transmitter and receiver behavior, i.e., when θ=γ=0 in Eq. **3**, there will be no correlation between the probability of producing misinformation $v_{f}$ and the probability of engagement among receivers).

## Finding Successful Transmitting Strategies.

Having defined a strategy space for transmitters and receivers and having described how a transmitter can unilaterally constrain the dynamics of the misinformation game (Eq. **3**), we now examine the dynamics of a receiver’s engagement strategy in response to a strategic transmitter (*SI Appendix*, Fig. S3). We study a single receiver who employs a noisy optimization process in an attempt to increase engagement with true stories (that is, to increase payoff given truth-seeking preferences; see *SI Appendix*, section 1.5). We focus on a myopic optimization process in which the receiver compares the payoff they gain under their current strategy to that under a randomly selected alternate strategy and tends to adopt the alternate strategy if it will increase their payoff, such that they adopt the new strategy, and discard the old, with probability $\pi_{i\to j}$ determined by a Fermi function $\pi_{i\to j}=\frac{1}{1+\exp[\sigma \left(w_{i}-w_{j}\right)]}$ where $w_{i}$ is the payoff received under strategy i, and σ determines the level of attention the player pays to their payoffs (*SI Appendix*, section 1.5). The case of a single transmitter and receiver corresponds to perfect microtargeting by the transmitter (i.e., the transmitter is able to promote different types of news to specific receivers in direct response to their engagement habits). However, our results also hold in the more realistic case where a transmitter is able to target their stories only at the level of a group of receivers (*SI Appendix*, section 3.6), and in the case where receivers interact with multiple transmitters (*SI Appendix*, section 3.8). In *SI Appendix*, section 1.4, we also consider the case where receivers are perfectly rational (28, 29), and the case where receiver behavior is the product of social learning (10, 30) (*SI Appendix*, section 3.3), where we find similar results as for the local optimization process.

We simulated strategy optimization for inattentive receivers ($a_{0}=0$ and σ=1) against $10^{8}$ randomly selected transmitter strategies r={α,β,γ,θ} who use feedback (Eq. **3**) as well as $10^{8}$ randomly selected transmitter strategies r={α,β,0,0} who do not use feedback. We measured the equilibrium probability with which the transmitter shares false stories with the receiver, $v_{f}$, and the probability with which the receiver engages with false stories (probability of engaging per false story) $v_{\mathrm{fc}}/v_{f}$. We also measured the probability with which the transmitter shares true stories with the receiver, $v_{t}=1-v_{f}$, and the probability with which the receiver engages with true stories (probability of engaging per true story) $v_{\mathrm{tc}}/v_{t}$ (*Materials and Methods*).

We define the most successful misinformation transmission strategies as those that i) shared misinformation more often than not, $v_{f}>0.5$ and ii) produced a misinformation engagement probability within the top 10% of all transmitter strategies considered (*Materials and Methods* and *SI Appendix*, Fig. S3). We consider alternate definitions, in which the minimum amount of misinformation shared varies, in *SI Appendix*, Fig. S4. Likewise, we define successful accurate news transmission strategies as those that produce $v_{t}>0.5$ along with true news engagement within the top 10% of all transmitter strategies considered. Note that accurate transmitters, when faced with inattentive and myopic readers whose incentives are unknown, may also share a mix of true and false stories as they attempt to induce engagement, just as misinformation sites may share accurate stories to draw readers in.

## Characterizing Successful Producer Strategies.

To quantify how transmission strategies shape engagement, we calculated the resulting correlation between story accuracy and receiver engagement. When feedback is present, the most successful accurate and misinformation dissemination strategies induce characteristic, and opposite, patterns of engagement among readers in our model: Successful misinformation sites induce higher reader engagement with each false story as well as greater overall engagement with false than true stories (*SI Appendix*, Fig. S3 and Table S2). This pattern arises even when we assume that receivers prefer accurate news over misinformation, so there is no inherent appeal of false stories.

To understand this phenomenon, we inspected the strategies of successful misinformation sites under our model. We find that all successful misinformation sites indeed use strategies that employ feedback in such a way as to enforce a positive correlation between engagement and misinformation transmission. A significant proportion (72%, *Materials and Methods*) use a type of responsive strategy that enforces $v_{f}\geq v_{\mathrm{fc}}+v_{\mathrm{tc}}$, i.e., successful misinformation site strategies tend to increase their false stories output rapidly in response to increased engagement. However, if engagement drops, they tend to increase their output of true stories (to draw the user back in). As a result, they “mash up” true and fake stories as engagement fluctuates over time.

The behavior of successful accurate news sites, which seek to generate engagement with true stories, shows the opposite pattern to successful misinformation sites. All successful accurate strategies enforce a negative correlation between engagement and misinformation transmission. A substantial proportion (56%, *Materials and Methods*) use a strategy that enforces $1-v_{f}\geq v_{\mathrm{fc}}+v_{\mathrm{tc}}$, i.e., successful strategies of sites seeking to promote accurate information tend to decrease their output of false stories rapidly in response to increased engagement, but may share more false stories when engagement is low (in a misguided attempt to draw readers back in). We also show that transmitter strategies generated through a process of co-optimization by transmitters and receivers, produces similar patterns (*SI Appendix*, section 3.3).

## Co-optimization of Transmitter and Receiver Strategies.

**Fig. 3.**
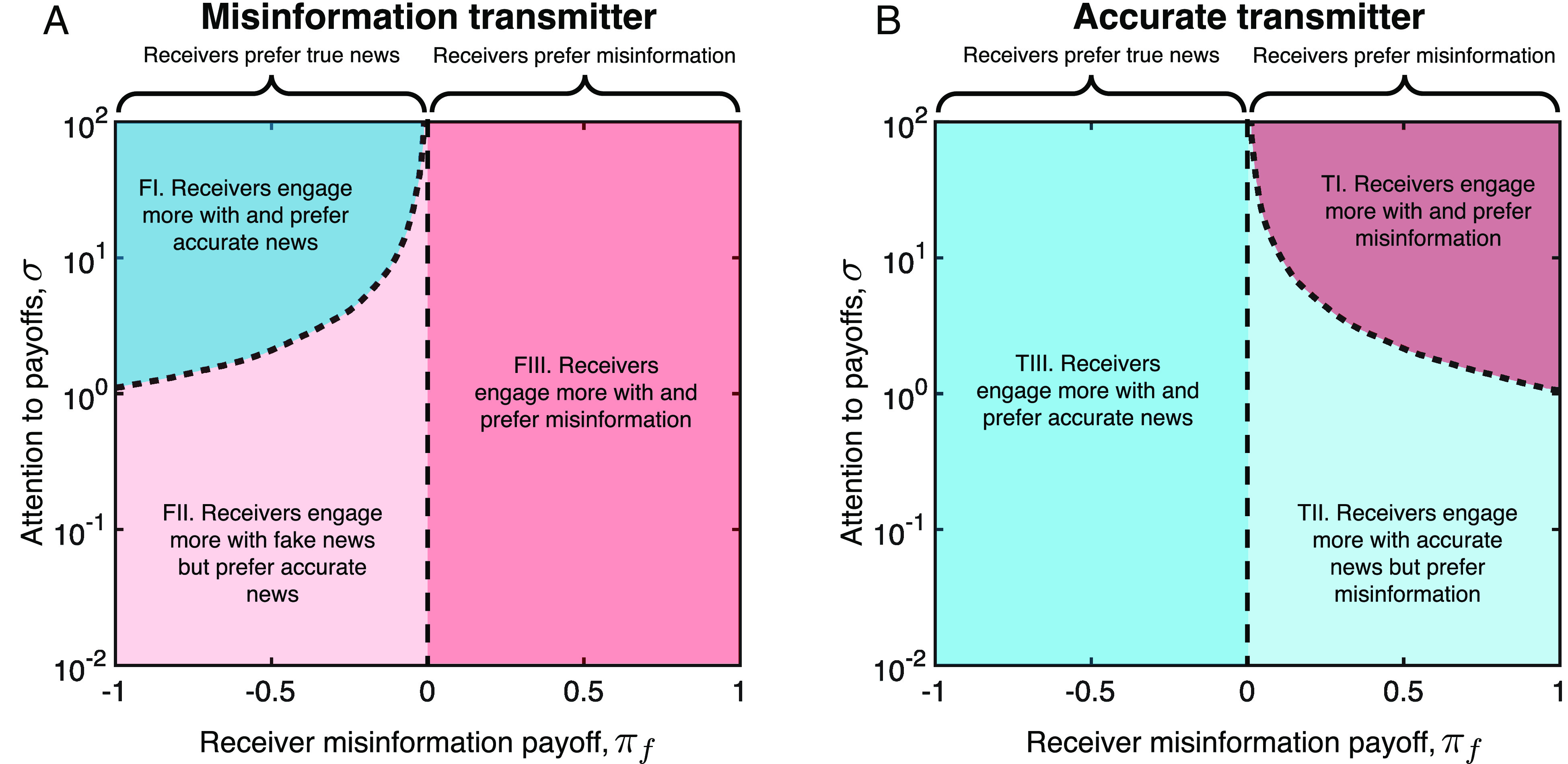
Transmitter strategy interacts with receiver preferences and attentiveness to determine engagement—(*A*) We calculated the engagement patterns among receivers optimizing under different preferences (where we have set $\pi_{t}=-\pi_{f}$), and different levels of attention to payoff, σ. We allow a misinformation transmitter ($b_{f}=1$ and $b_{t}=0$) and a receiver to co-optimize and identify the regions in which receivers have a higher probability of engaging with accurate vs. fake stories. In region F1, receivers prefer true news and attention to payoff is high, so that receivers engage more with accurate than with fake stories. In region F2, receivers prefer accurate news but attention to payoff is low, and receivers engage more with fake stories. In region F3, receivers prefer fake stories and engage more with fake stories regardless of their level of attention to payoffs. (*B*) We carried out the same procedure as in panel *A*, for an accurate transmitter ($b_{f}=0$ and $b_{t}=1$). In region TI, receivers prefer misinformation and attention to payoffs is high, so that receivers engage more with fake than with accurate stories. In region T2, receivers prefer misinformation but attention to payoffs is low and receivers engage more with accurate stories. In region T3, receivers prefer accurate stories and engage more with accurate stories regardless of their level of attention to payoffs. In all cases, receiver strategic exploration was local, with $a_{0}=a_{1}=0$ (equivalent results when $a_{0}>0$ and $a_{1}>0$ are shown in *SI Appendix*, Figs. S13 and S17). Transmitter attention to payoffs set at σ=100. Optimization occurred over $10^{4}$ time-steps using ensembles of $10^{3}$ replicates for each value of $\left\{\pi_{f},\sigma \right\}$ (*Materials and Methods*).

Next, we explored the dynamics of reader engagement under different reader preferences, $\pi_{t}$ and $\pi_{f}$, and different levels of reader attention to payoff, σ. We allowed readers to optimize their engagement strategies when interacting with misinformation and accurate transmitters who also seek to optimize their transmission strategy (*Materials and Methods*). We find three distinct regions for engagement patterns against both misinformation and accurate transmitters (Fig. 3). We begin by considering the case of receivers interacting with misinformation transmitters. When receivers prefer accurate information and are highly attentive (region F1), they engage more with accurate news stories. When receivers who prefer accurate news are at least somewhat inattentive (region F2); however, the strategic behavior of the transmitter causes the receivers to engage more with misinformation despite their preference for true news. When receivers who prefer inaccurate information interact with misinformation transmitters, there is no mismatch between transmitter and receiver preferences and receivers always engage more with misinformation than with accurate stores (region F3). When considering the case of receivers interacting with accurate transmitters, we find a symmetric set of outcomes (regions T1 to T3).

Critically, there are two large regions (F2 and T2), which produce patterns of receiver engagement that do not reflect the receiver’s own preferences. In these regions—where receivers are not highly attentive and have preferences that are misaligned with those of the transmitter—it is the transmitter’s preferences, and their use of responsive strategies to implement those preferences, that determine engagement among receivers. Our predictions for these regions are distinct from the predictions of previously proposed theories for explaining engagement with misinformation (15, 24, 27), which allow only for regions F1/T1 and F3/T3 (in which receivers’ preferences are aligned with their engagement patterns; *SI Appendix*, section 2).

We demonstrate the robustness of our theoretical results in *SI Appendix*, section 3. We show that the existence of region F2/T2 holds when receivers can choose between multiple different news sources (*SI Appendix*, section 3.8), in a scenario where consumers respond to a “pool” of news from multiple sources. We also explore the impact of microtargeting and receiver attention to accuracy. We find that when microtargeting is low, the difference in engagement between false versus true stories declines. Thus, the ability to target news stories at specific receivers, either by news sites directly or by social media algorithms, can exacerbate the ability of misinformation sources to drive engagement with false stories despite reader preferences for accuracy (*SI Appendix*, Fig. S11). We also explored the impact of two other forms of receiver attention: i) attention to and/or prior knowledge of story accuracy, $a_{0}$, ii) memory of past interactions when deciding to engage with a news source, $a_{1}$ (*Materials and Methods* and *SI Appendix*, section 2). We find that increasing prior knowledge of or attention to headline accuracy reduces both the engagement probability per false story and the overall engagement with false stories under an optimization process (*SI Appendix*, Fig. S15–S17).

We also show that transmitters who seek only to maximize engagement among receivers ($b_{f}=b_{t}$), but make different assumptions about the type of news those receivers prefer, can inadvertently shape the patterns of engagement among at least somewhat inattentive readers. Transmitters who assume receivers prefer misinformation (e.g., because it is more novel) employ strategies that seek to reinforce increased engagement by increasing the amount of false stories they share in response (*SI Appendix*, section 1). As a result, false stories will get more engagement than true stories, which will then reinforce the transmitter’s initial assumption (even if it is wrong), creating a self-fulfilling prophecy. Reciprocally, transmitters who assume receivers prefer true news employ strategies that seek to reinforce increased engagement by increasing the amount of true news they share in response—and generate a pattern of increased engagement with true news. Transmitters who make no assumption about receivers preferences (and hence try out all possible strategies without bias) and transmitters who assume receivers prefer true news, both produce greater engagement with true than with false stories (*SI Appendix*, Fig. S5).

## Empirical Patterns of Misinformation Engagement.

Our analysis of the misinformation game presented above identifies three classes of engagement dynamics with misinformation: The patterns of engagement among those who read misinformation sites fall into either region F1, F2, or F3 of Fig. 3*A*. Only if readers prefer accurate information and are highly attentive to their patterns of news consumption will they engage more with accurate stories than with false stories from misinformation sites (region FI). However, if readers prefer accurate information, but are not highly attentive to their patterns of news consumption, they will fall into region F2—where misinformation sites can induce high engagement with fake stories by using feedback, despite readers’ preference for veracity (Eq. **2**). Finally, if readers prefer inaccurate information, they will fall into region F3, where their preference straightforwardly leads them to engage more with inaccurate information. As shown in Fig. 3*B*, the converse pattern holds for mainstream news sites that aim to spread accurate information. We now present two sets of experiments designed to determine which region(s) reflect the actual dynamics of misinformation engagement observed empirically.

First, we ask whether inaccurate articles generate more or less engagement than accurate articles—and whether this correlation between engagement and perceived accuracy differs for content published by misinformation versus mainstream sites. To do so, we used data on Facebook engagement with news from publishers that prior work has determined to be either misinformation or mainstream outlets (31). We sampled a total of 1,000 articles from 40 outlets (20 misinformation, 20 mainstream). We selected the 25 most recently available news stories for each site from the Crowdtangle database (*Materials and Methods*), providing a snapshot of the output from each site and avoiding selecting on the dependent variable by only analyzing high-engagement articles (32). For each story, we retrieved Facebook engagement ratings from Crowdtangle (25) (*Materials and Methods*). We also assessed each story’s perceived accuracy by recruiting 1,000 American participants from Amazon Mechanical Turk to rate the accuracy of 20 headlines (yielding a total of 20,000 accuracy ratings), which has been shown to produce good agreement with the ratings of professional fact-checkers via the wisdom of crowds (33).

**Fig. 4.**
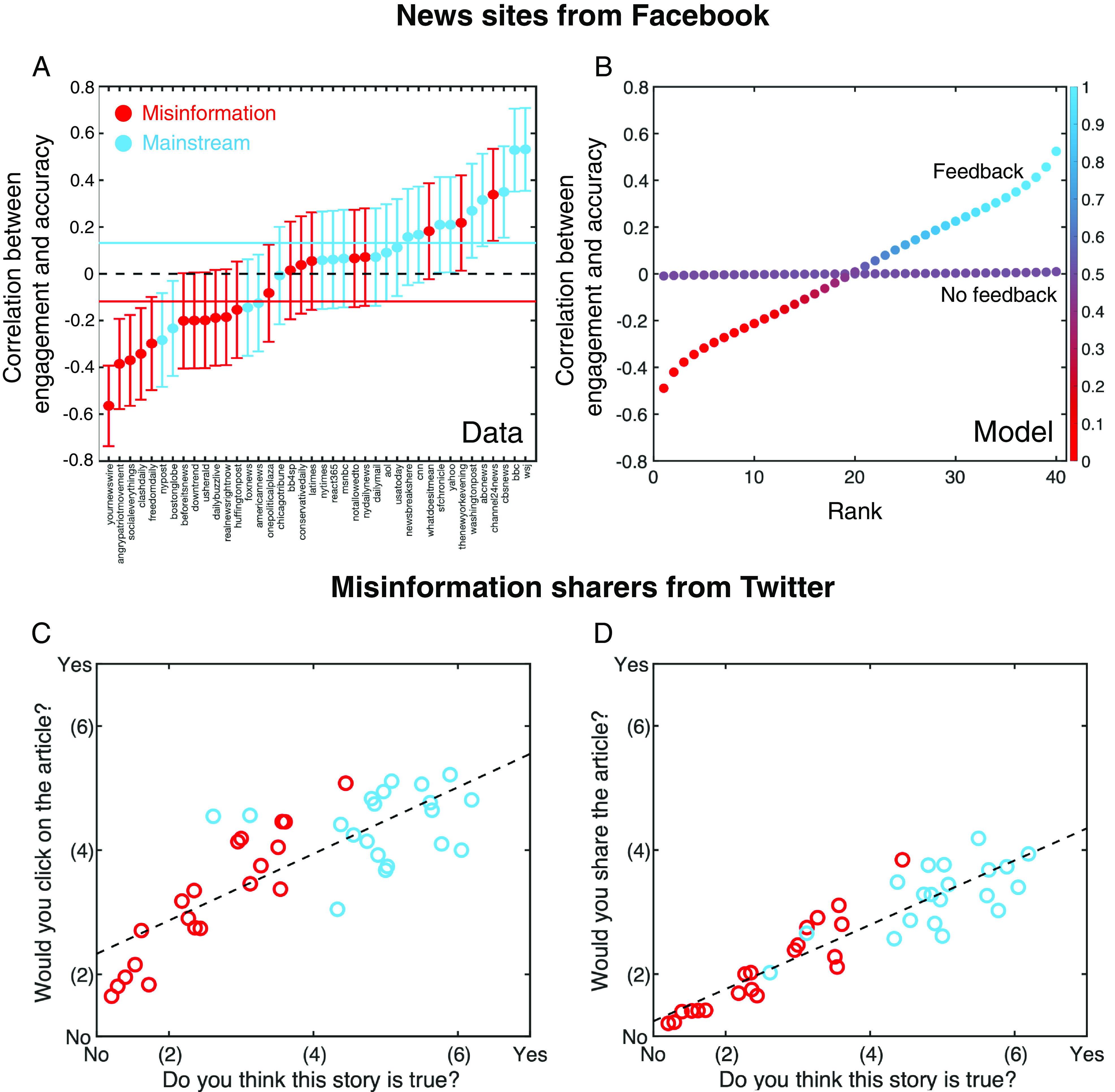
Less plausible stories receive more engagement on misinformation sites —even though misinformation sharers prefer to share accurate news. We selected 20 mainstream and 20 misinformation sites identified in previous studies of misinformation (31) (see *SI Appendix*, Table S5 for list). Using Crowdtangle we selected the most recent 25 news stories for which engagement data on Facebook was available (*Materials and Methods*). We then recruited American subjects from Amazon Mechanical Turk to assess headline accuracy (20 ratings per headline). (*A*) Regression coefficient between accuracy and $\log_{10}$-engagement for mainstream news sites (blue) and misinformation sites (red). There is a significant positive correlation between accuracy and engagement across mainstream sites [Fisher’s combined test, P=0.004; Random effects meta-analysis (34, 35), P=0.005, standardized average correlation coefficient—blue line—μ=0.13; *Materials and Methods* and *SI Appendix*, section 3]. However, there is a significant negative correlation between accuracy and engagement across misinformation sites (Fisher’s combined test, P=0.005; Random effects meta-analysis, P=0.013; standardized average correlation coefficient—red line—μ=−0.11; *Materials and Methods* and *SI Appendix*, section 3). (*B*) Computational experiment, selecting $10^{4}$ replicates of 20 successful accurate transmitters and 20 successful misinformation transmitters as described in *SI Appendix*, Fig. S3. For each replicate, we rank ordered the regression coefficients from lowest to highest. Shown is the average regression coefficient in each rank with and without feedback. Colors indicate the proportion of sites in that position that are accurate (blue) or misinformation (red). When feedback is present, the qualitative pattern observed empirically is reproduced by the model. As in Fig. 3, computational results are for local receiver strategic exploration (*SI Appendix*, section 1.5) with transmitter error rates of 0.3 (*Materials and Methods*). (*C*) We selected 20 mainstream (blue) and 20 misinformation (red) stories identified in previous studies of misinformation (31). We then recruited a sample of 119 participants directly from Twitter who had previously shared misinformation to assess the accuracy of 10 headlines of each type. Each participant was then asked to rate their willingness to click on and (*D*) share the article associated with the headline. We observe a significant positive association between accuracy and willingness to click (β=.491,z=11.37,P<0.001) or share (β=.640,z=14.01,P<0.001). As per our pre-registered analysis plans, all analyses use linear regression conducted at the rating level, with all variables z-scored and using robust SEs clustered on subject and headline. For visualization, we average ratings across subjects for each headline and plot headline-level associations.

Before turning to the key question of the correlation between engagement and perceived accuracy, we begin by noting an expected basic descriptive pattern: mainstream news sites, which we assume seek to promote accurate information, tend to share headlines with higher accuracy ratings than misinformation sites (P<0.001, Fig. 4*A*). Importantly, however, both mainstream and misinformation sites show wide variation in perceived headline accuracy. Thus, there is substantial overlap in plausibility between the content produced by the two kinds of sites, with many articles from misinformation sites being rated as more accurate than many articles from mainstream sites.

How, then, does engagement on Facebook vary across this range of perceived accuracy? The answer is strikingly different for mainstream versus misinformation sites. We find a significant negative correlation between engagement and perceived accuracy for articles from misinformation sites (P=0.005), whereas we find a significant positive correlation for articles from mainstream sites (P=0.004).

This pattern rules out the case in which all readers are highly attentive and prefer accurate news, which would lead to more engagement for more accurate articles regardless of publisher type; as well as the case in which all readers are highly attentive and prefer inaccurate news, which would lead to less engagement for more accurate articles regardless of publisher type. Instead, the observed pattern is consistent with readers who belong in either region F2/T2 or region F3/T3 for both misinformation and mainstream sites. That is, readers of misinformation sites either prefer less accurate information (region F3), or they prefer accurate information but are sufficiently inattentive that their engagement habits can be shaped by misinformation transmitters (region F2); and readers of mainstream sites either prefer accurate information (region T3), or they prefer inaccurate information but are sufficiently inattentive that their engagement habits can be shaped by mainstream transmitters (region T2). In order to differentiate between these possibilities, we conducted a second set of experiments to measure users’ preferences for engaging with accurate versus inaccurate news.

## Empirical Patterns of Receiver Engagement Preference.

We empirically determined the preferences of readers who engage with misinformation sites versus mainstream news sites through a set of preregistered survey experiments (*Materials and Methods*). First, we recruited 511 members of the general population via Lucid (see Methods), of whom 124 indicated regularly using one or more fake sites; and second, we recruited 119 participants directly from Twitter who had actually shared links to misinformation sites previously (behaviorally demonstrating their engagement with such sites). Each participant was shown a series of true and false headlines and for each was asked to rate the headline’s accuracy and indicate how likely they would be to click on and to share the story if they saw it online. If we find that subjects who use misinformation sites prefer to engage with content they perceived as inaccurate, this would suggest that reader preference is driving the greater engagement rates for more inaccurate headlines observed in Fig. 4*A* (i.e., suggests we are in region F3 in Fig. 3*A*). If, on the other hand, those who use misinformation sites actually prefer to engage with content they perceive as accurate, this is consistent with the interpretation that reader preferences alone are not driving the pattern of higher engagement for more inaccurate content observed in Fig. 4*A*—rather the strategies of producers may cause inaccurate content to receive more engagement (region F2 in Fig. 3*A*).

The observed preferences are strikingly consistent across the different types of users. In all cases, there is a significant positive correlation between perceived accuracy and willingness of readers to click or share an article (P<0.001 for both outcomes across all three groups, Fig. 4 and *SI Appendix*, Figs. S21–23). This holds for subjects recruited from Twitter who had previously shared misinformation, as well as those recruited from the general population regardless of their self-reported use of misinformation sites. The results are qualitatively equivalent when using objective accuracy (as measured by professional fact-checkers; P<0.001 for both outcomes across all three groups), and our findings continue to hold under replication in a Supplemental Experiment conducted with a general population sample in which participants were only asked their willingness to click and share each headline, without first rating the headline’s accuracy; see *SI Appendix*, section 4).

Thus, our empirical results indicate that the pattern of reader engagement with misinformation sites falls in region F2: the observed pattern reflects the transmitter’s desire to generate high engagement with misinformation, and not a consumer preference for engaging with inaccurate news. For mainstream sites, the pattern of reader engagement with mainstream sites falls into region T3, in which the preferences of the reader and transmitter are aligned (both seeking high engagement with accurate news).

Taken together, these empirical results imply that reader preferences cannot be reliably inferred from patterns of reader engagement. Rather they suggest a more complicated interaction, in which reader engagement with news may result from a feedback loop generated by misinformation sites acting on not fully attentive readers who prefer accurate news—but nonetheless engage more with inaccurate news due to the transmitter’s behavioral strategy.

## Discussion

Understanding why people engage with misinformation has become increasingly important. Widespread belief in false information can destabilize democratic institutions, fuel populist movements and polarization, and undermine public health efforts, as seen during the COVID-19 pandemic. Here, we develop formal game theoretic models which shed light light on why misinformation spreads. We developed a framework to study the strategic interaction between readers and news sources, and combined this with empirical analyses of patterns of engagement with misinformation and mainstream news sites, as well as the expressed preferences of their readers. Our theoretical analysis reveals two key features of the dynamics of consumer engagement with misinformation. First, misinformation sites that are likely to be successful in producing high overall levels of engagement among readers will tend to produce patterns of engagement in which the false stories receive more engagement than true stories receive, even if true stories are preferred over false stories by their readers (region F2 of Fig. 3*A*). In contrast, mainstream sites that seek to promote engagement with accurate stories should tend to produce the opposite engagement pattern among their readers, unless those readers are highly attentive and actively prefer to engage with misinformation (Fig. 3*B*). Second, a misinformation site committed to pushing as much false information as possible into the information ecosystem should—when faced with readers who prefer accurate stories—use responsive strategy and, as a result, publish a substantial amount of true news alongside the false.

While our model is a highly simplified description of the interactions that occur in complex online information ecosystems, it illustrates the dangers of drawing conclusions about consumer preferences without accounting for the supply side of news. Our results cast a different light on previous findings that false stories receive more engagement than true stories, which has been interpreted as reflecting a consumer preference for novel but false information (27). This observation stands in contrast to other studies, which found that true stories receive as much or more engagement than false stories (7, 11). Our results suggest that these different patterns may be explained by the different sources of news examined in the different studies. Among claims that have been fact-checked by Snopes (largely coming from misinformation sources)—as focused on in ref. 27—we might expect less accurate news to get more engagement due to the behavior of its suppliers. Conversely, when news come from a more balanced set of sources, as in refs. 7 and 11, we would not expect the same pattern to emerge. Of course, our findings do not rule out the possibility that novelty may drive engagement with misinformation (27), but our work shows that high levels of misinformation engagement can arise even in the absence of intrinsic novelty appeal or motivated reasoning, and especially when transmitters behave in a highly responsive manner by microtargeting their content (36). Our results also help to explain why misinformation sites publish so much content that is actually quite plausible (*SI Appendix*, Fig. S21). To garner engagement, responsive misinformation-spreading strategies must provide enough accurate-seeming content to avoid alienating their readers, who prefer to engage with accurate news. An important avenue for future work will be to explore the impact of such producers’ strategies on the dynamics of producer competition in a competitive market.

The idea that misinformation sites can successfully induce readers into engaging with false stories has important practical implications. First, our findings suggest an approach to identifying outlets that are seeking to spread misinformation: examining the relationship between articles’ engagement and perceived accuracy. Outlets from which particularly implausible articles generate particularly high levels of engagement may be aiding the spread of misinformation by employing responsive transmission strategies. Observing such a relationship could be used as a signal that an outlet is intent on spreading misinformation, or making faulty assumptions about the preferences of readers. Similarly, if such a relationship is observed in the patterns of engagement with inaccurate stories on a particular social media platform, this may suggest the platform uses responsive strategies to promote misinformation to users. Second, the tendency to share misinformation can become self-reinforcing on the part of transmitters: sites that seek to spread misinformation using responsive strategies can create the incorrect impression that readers prefer misinformation. This pattern, if taken at face value, may then lead to increased misinformation production by sites that do not have any particular agenda beyond simply maximizing engagement—even when such sites would actually maximize engagement by publishing true articles. Third, we find that this apparent preference for misinformation can be reversed by encouraging readers to pay attention to key aspects of their news consumption habits. This observation reinforces the importance of (in)attention in combating misinformation.

In sum, we have demonstrated the importance of the feedback loop between news publishers and readers. We have shown that focusing only on reader behavior can lead to incorrect conclusions about readers’ preferences, while considering publisher strategies can resolve apparent contradictions in the literature, and can highlight possible directions for combating misinformation. News consumption does not occur in a vacuum, and to understand the dynamics of misinformation it is essential to explore supply as well as demand.

## Materials and Methods

### Simulations.

We performed simulations to determine the most successful transmitter strategies for fake and mainstream sites. We selected a transmitter strategy r={α,β,γ,θ} (Eq. **2**) with each parameter drawn uniformly from the interval required to produce a viable strategy. We initialized a receiver with a strategy that does not engage with any news from the transmitter, i.e., q={0,0,0,0} (Eq. **1**) which was then allowed to update under a local optimization process (*SI Appendix*, section 1). For each set of receiver and transmitter strategies, we calculated the stationary distribution for transmission and engagement of news stories $v=\{v_{\mathrm{tc}},v_{\mathrm{tn}},v_{\mathrm{fc}},v_{\mathrm{fn}}\}$ under the iterated misinformation game. For a single receiver, the stationary distribution can be found explicitly. For a larger population of receivers we simulated $10^{4}$ rounds of the game, which allowed us to estimate the stationary distribution numerically. In all simulations we assume that both transmitters and receivers experience execution errors (37) with probability $\varepsilon =10^{-3}$. Finally, we assume a “perception error” among transmitters, in which they incorrectly label false stories as true, or vice versa, of η=0.3. Transmitter strategies without feedback were produced in the same way, with the constraint θ=γ=0.

We used the stationary distribution v to calculate receiver payoffs at equilibrium. We then allowed receiver strategies to update under a local optimization process (see *SI Appendix*, section 1.5 for further details). After a burn in period of $10^{4}$ update events, we measured the average engagement and engagement probability of receivers over an additional $10^{4}$ update events.

Simulations in which transmitters and/or multiple receivers co-optimize followed the same procedure as for a single receiver and transmitter, with all players engaging in the local optimization process. Region plots (Fig. 3 and *SI Appendix*, section 3) were produced by breaking the parameter space into a 100×100 grid and co-optimizing $10^{3}$ replicates at each point.

Transmitters were identified as employing extortion strategies, $r_{+}^{*}$ or $r_{-}^{*}$, if their strategy lay within a Δ-neighborhood of a “true” extortion strategy (38, 39), with Δ=0.05. Significant over-representation of extortion strategies among the most successful fake and mainstream sites (i.e., those strategies that successfully produce engagement with fake and true stories respectively) was then determined by comparing the prevalence of the strategy in our data to the null distribution, i.e., the probability with which such strategies are randomly drawn. Significance level quoted is P<0.01.

### Modeling Experiments.

In order to assess the role of transmitter feedback in producing empirical patterns of engagement with different types of news site (*SI Appendix* Fig. S3 and Fig. 4*B*), we ran simulation experiments. We randomly selected successful transmitter strategies and their corresponding receiver strategy. For each transmitter, we generated a sequence of 20 news stories according to their strategy, and calculated the probability of engagement among a population of $10^{5}$ receivers for each story based on the associated receiver strategy. This produced a single simulated experiment, and we calculated the regression coefficient for the standardized engagement rate against the perceived accuracy of the story under perception error η (*SI Appendix*, Fig. S3). This process was repeated $4\times 10^{5}$ times to produce a distribution of accuracy and regression coefficients (*SI Appendix*, Fig. S3), for both accurate and misinformation transmitters, with and without transmitter feedback.

Next, we simulated a rank ordering of regression coefficients (main text Fig. 4*B*) by randomly drawing 20 accurate transmitter strategies and 20 misinformation transmitter strategies, and calculating their simulated regression coefficient as described above. We then ordered all 40 strategies from lowest to highest to produce a ranking. We repeated this procedure $10^{4}$ times to produce an average regression coefficient in each ranked position, for transmitter strategies with and without feedback, as shown in Fig. 4*B*.

### Experiment A: Empirical Patterns of Misinformation Engagement.

We asked participants to evaluate the accuracy of 1,000 articles across 40 mainstream and misinformation sites, gathered from Crowdtangle, a tool for monitoring engagement on Social Media). The study was approved by MIT institutional review board (IRB) (protocol 1806400195). Informed consent was provided at the beginning of the study.

#### Participants.

From 22 to 29 November 2020, we recruited 1,000 participants from Amazon Mechanical Turk who met the following three criteria: located in the United States, more than 100 studies completed on the platform, and more than 95% of them approved. A total of 1,027 participants initiated the study but 27 did not complete the evaluation task and were excluded. The sample included 576 males and 424 females, with a mean age of 38.49 y (min. 19; max. 96). Median completion time was 4 min and 55 s.

#### Materials.

We used Crowdtangle to gather the 25 most recently available news stories from 40 media outlets (i.e., 1,000 articles; 500 posted by 20 misinformation sites and 500 posted by 20 mainstream sites), along with the headline, lede, date of publication, link, and level of engagement. See *SI Appendix*, section 3 for additional details. We then used this data to present the participants outlined above with 20 article headlines and ledes, drawn randomly from within one of the two media outlet subsets, and asked them to assess the accuracy of the information they were faced with. Specifically, they were asked “Do you think this story is true?,” to which they responded on a seven-point scale from “Definitely NO” to “Definitely YES.” The study concluded with seven demographic questions (age, gender, education, political conservativeness on social and economic issues, political position, and political preference) and a section to leave comments.

### Experiment B: Empirical Patterns of Receiver Preference (Lucid).

We asked participants to assess the accuracy, likelihood of sharing, and likelihood of clicking on 40 headlines. We then asked which domains they regularly use for news (*SI Appendix*, Figs. S22 and S23). The study was approved by MIT institutional review board (protocol 1806400195). Informed consent was provided at the beginning of the study.

#### Participants.

We recruited American participants via Lucid (a widely used platform for recruiting participants for online experiments) from March 15 to 25, 2022, quota-matched to the national distribution on age, gender, ethnicity, and geographic region. In accordance with our preregistration (90974) we excluded: 1 participant located outside the United States, 188 who failed one or two of the trivial attention checks at the study outset, and 79 who reported not having at least one social media account. We also excluded 21 participants who did not declare using at least one of the 60 listed domains for news (as these participants cannot be classified as users of either misinformation sites or nonmisinformation sites), leaving a final sample of 511 subjects. The sample included 237 males and 259 females, with a mean age of 47.01 y (min. 18; max. 87). Median completion time was 10 min and 39 s.

#### Materials.

We identified a pool of 40 news “cards” (i.e., representations of Facebook posts with an image, headline, and a source; balanced on veracity and partisan lean) and a list of 60 domains regularly used for news 20 identified as mainstream, 20 as hyper-partisan, and as 20 fake by Pennycook and Rand, 2019 (31). We then asked participants to : i) evaluate 20 cards, drawn randomly from the set of 40, on the accuracy (i.e., “Do you think this story is true?,” seven-point scale from “Definitely NO” to “Definitely YES”), likelihood of sharing (i.e., “If you were to see the above headline online, would you share it?,” seven-point scale from “Definitely NO” to “Definitely YES”), and likelihood of clicking (i.e., “If you were to see the above headline online, would you click on it to read the article?,” seven-point scale from “Definitely NO” to “Definitely YES”) of the information presented; ii) select which of the 60 domains they regularly use for news (with this information, we classified participants as misinformation media users if they selected at least one domain pre-identified as a misinformation outlet). The study concluded with a list of 20 exploratory items.

### Experiment C: Empirical Patterns of Receiver Preference (Twitter).

We recruited participants for Experiment C through an advertisement campaign on Twitter conducted between April 22 and April 29, 2022. We created a set of 24 ad creatives that paired different images with the text “We want to know your opinion! Take a 5-min survey about the news. Just click here” and targeted a custom audience of all the Twitter users who had previously engaged with content from any of the 20 misinformation sites identified by Pennycook and Rand (31). Upon clicking on the advertisement, participants were redirected to a survey asking them to assess the accuracy, likelihood of sharing, and likelihood of clicking on the same 40 headlines used in Experiment B. The study was approved by MIT institutional review board (protocol 1806400195). Informed consent was provided at the beginning of the study.

#### Participants.

From April 22 to 29, 2022, 206 participants entered the survey. Of these, we excluded 87 who failed at least one of the two attention checks of our study. The final sample of 119 participants included 60 males, 53 females, and six participants who preferred not to answer, with a mean age of 57.81 y (min. 28; max. 79). Median completion time was 6 min and 14 s.

#### Materials.

We use the same pool of 40 politically and veracity-balanced news “cards” from Experiment B. We also asked participants to evaluate cards based on accuracy, likelihood of sharing, and likelihood of clicking, just like in Experiment B. The only difference between the questionnaires of the two experiments was that we asked participants to evaluate 10 cards instead of 20, that we did not ask them to select what domains they use for news, and that we concluded with a list of 10 exploratory items instead of 20.

## Supplementary Material

Appendix 01 (PDF)

## Data Availability

Survey responses and engagement rates data have been deposited in GitHub (https://github.com/al-cibiades/Coercive-logic-of-fake-news) (40).
