# Supplementary material for: The distorting effects of producer strategies: Why engagement does not reveal consumer preferences for misinformation
Source: Proc Natl Acad Sci U S A. 2024 Feb 27;121(10):e2315195121. doi: 10.1073/pnas.2315195121 (PMC10927527; doi:10.1073/pnas.2315195121)
Supplement: Supplementary file 1 — Appendix 01 (PDF) [file pnas.2315195121.sapp.pdf]

The distorting effects of producer strategies: Why engagement does not reveal consumer preferences for misinformation - Supplementary Information

Alexander J. Stewart[1*], Antonio A. Arechar[2], David G. Rand[2], and Joshua B. Plotkin[3]

[1]*School of Mathematics and Statistics, University of St Andrews, St Andrews, KY16 9SS, United Kingdom*
[2]*Sloan School of Management, MIT, Cambridge, MA, USA*
[3]*Department of Biology, University of Pennsylvania, Philadelphia, PA, USA*
[*]*ajs50@st-andrews.ac.uk*

Contents

1 Analysis of the misinformation game 5

1.1 Overview and background . . . 5

1.2 Dynamics of play in the infinitely iterated misinformation game . . . 7

1.3 Stationary distribution . . . 8

1.4 Responsive strategies . . . 11

1.5 Necessary and sufficient conditions for extortion under linear transmitter strategies . 13

1.6 Nash equilibria in the misinformation game . . . 15

1.7 Modelling local optimization . . . 18

1.8 Time series for linear and non-linear transmitter strategies . . . 18

1.9 Modelling optimization through social learning . . . 19

1.10 Feedback and receiver engagement . . . 21

2 Comparison to previous models 23

2.1 Vosoughi et. al. 2018 . . . 23

2.2 Pennycook et. al 2021 . . . 23

2.3 Allcott and Gentzkow 2017 . . . 24

3 Further analysis and extensions of the model 26

3.1 Identifying successful transmitter strategies . . . 26

3.2 Co-optimizing transmitters and receivers . . . 31

3.3 Comparison of most successful transmitters with co-evolved transmitters . . . 33

3.4 Impact of absolute transmitter payoff . . . 34

3.5 The effect of receiver learning model choice . . . 36

3.6 The effect of receiver group size and transmitter microtargetting . . . 37

3.7 The effect of different types of receiver attention . . . 40

3.8 The effect of multiple news transmitters on consumer behavior . . . 47

3.9 The effect of supply and demand . . . 49

4 Supplementary information on experiments **51**

4.1 Headline selection and engagement data 51

4.2 Meta-analysis 51

4.3 Experiment A: Empirical patterns of misinformation engagement (MTurk). 53

4.4 Experiment B: Empirical patterns of receiver preference, with accuracy elicited (Lucid). 55

4.5 Supplemental Experiment: Empirical patterns of receiver preference, without accuracy elicited (Lucid). 55

4.6 Breakdown of the demographic and political leanings of our participants 58

4.7 Robustness of empirical results to partisanship 60

4.8 Experimental interface 62

This supplement is broken into four sections. In the first section we present a formal analysis of the misinformation game, along with additional details of the modelling framework used in simulations. In the second section we place our approach in context of three prior models of bias and misinformation in news production. In the third section we provide a comprehensive set of additional simulations, including robustness checks of our findings to alternate model formulations and assumptions. In the fourth and final section we present details of additional experiments and meta-analysis to supplement those presented in the main text.

1 Analysis of the misinformation game

1.1 Overview and background

In this section we provide an analysis of the misinformation game, which, in its simplest form, is conceived as an infinitely repeated game between a receiver and a transmitter. The stage game payoff matrix for the misinformation game is described in Table S1. Alongside the payoff matrix we also show the game tree for a single round of play, which emphasises the fact that, in our game, a transmitter decides what to share, then a receiver decides whether to consume.

Our analysis draws on previous work originating in the study of "zero-determinant" strategies [11, 14, 13, 8, 1], in which the approach is to consider how the iterated game strategy of one player constrains the available payoffs of their opponent. This type of analysis is particularly useful if we wish to understand how the strategy of the first player shapes the behavior of their opponent while that opponent tries to optimise. In the context of the misinformation game, we are specifically interested in how the transmitter shapes the behaviour of the receiver.

In order to perform our analysis we make a number of simplifying assumptions. As in previous work [11, 14, 13, 8, 1] we focus on "memory-1" strategies for transmitters. Our analysis does not require any assumption about the memory of receivers, although we do of course make assumptions about receiver strategies in simulations (i.e. in Section 3). The use of a memory-1 transmitter strategy means that the transmitter conditions their behavior at a given round of the infinitely repeated game on the outcome of the preceding round. We assume that both players are subject to "execution errors" such that, occasionally, transmitters may mistakenly share a false (or true) story and receivers may mistakenly consume (or fail to consume) what the receiver shared. This allows us to calculate the stationary distribution for the iterated game. A given round of the game has four possible outcomes, (tc) [transmitter shares true news and the receiver consumes it], (tn) [transmitter shares true news and the receiver does not consume it], (fc) [transmitter shares fake news and the receiver consumes it] and (fn) [transmitter shares fake news and the receiver does not consume it]. The stationary distribution we calculate describes the equilibrium rates of these four outcomes in the infinitely iterated game. The basic question we then address is whether the

Transmitter

Receiver		Transmit true	Transmit fake
	Consume	(π_t, b_t)	(π_f, b_f)
	Do not consume	(0, 0)	(0, 0)

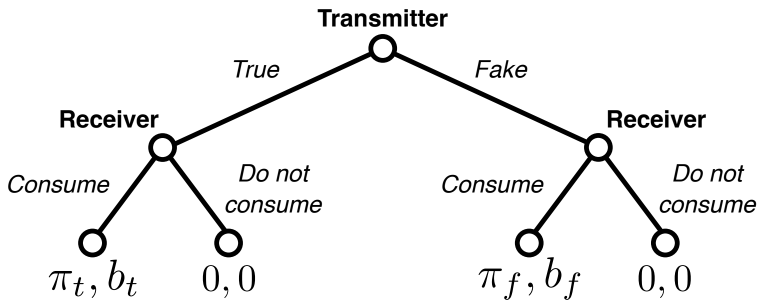

Table S1: **Stage game payoff matrix (top) and game tree for the stage game (bottom)**

transmitter can choose a fixed strategy such that a receiver, who seeks true news stories and who tries to optimize their payoff over time, will in fact end up consuming more fake news than true. In order to answer this, we look for transmitter strategies that ensure that greater consumption by receivers is positively correlated with fake news production. We then use simulations to show that such a transmitter strategy does indeed lead to receivers consuming fake stories at a greater rate than true stories. In this section we focus on the details of our formal misinformation game model, and carry out the analysis sketched above, i.e. we identify memory-1 transmitter strategies which ensure that greater consumption by receivers is positively correlated with fake news production.

In Section 3 we focus on the use of simulations to test the analytic results of Section 1, and to look at more complex scenarios, including when there are many transmitters or many receivers, and in which the "memory-1" assumption is violated. We also consider scenarios in which transmitters try to ensure receivers consume true news stories, and receivers prefer misinformation. Finally, we consider what happens when both receivers and transmitters try to optimise their behavior

simultaneously.

1.2 Dynamics of play in the infinitely iterated misinformation game

To begin our analysis we write down a recursion relation for the state of the game at round $j+1$ given the state at round j, in a scenario where a single memory-1 transmitter is interacting with N receivers using arbitrary (i.e. not necessarily memory-1) strategies. We assume that the payoffs to the transmitter are just the sum of their pairwise interactions as described in Table S1.

We consider a transmitter strategy $\mathbf{r}$ as described by Eq. 1 in the main text. To aid the reader we have reproduced Eq 1 below (without any equation number):

$$\begin{aligned} r_{kt} &= \alpha \quad + \sum_l \gamma_l \left(\tfrac{k}{N}\right)^l \\ r_{kf} &= \beta \quad + \sum_l \theta_l \left(\tfrac{k}{N}\right)^l . \end{aligned}$$

Here the probability of a transmitter sharing true news is r_{ki}, where the last piece of news shared was consumed by k receivers and was of type i. The possible types of news are true (t) or fake (f), so that $i \in \{t, f\}$, and in a population of N receivers the amount of engagement is $k \in [0, N]$. The transmitter strategy space we consider is thus memory-1. The transmitter strategy is described here in the most general form we consider i.e. as a power series in (k/N), where l indexes the polynomial terms and γ_l and θ_l are the associated coefficients. What we will show in the next sub-section is that a transmitter using a strategy of this type unilaterally enforces a linear relationship between receiver engagement and the amount of misinformation shared by the transmitter. This relationship is the one given by Eq. 3 in the main text.

We encode the state of the repeated game in round j as v_{ki}^{j} where k is the number of receivers who engage in round j and i is the type of news shared by the transmitter, $i \in \{t, f\}$. The probability that the game is in state v_{ki}^{j+1} at round $j+1$ is then given by[1]

[1]Note that equation numbers in the supplement follow on from the main text

$$v_{kt}^{j+1} = \sum_{m=0}^{N} \sum_{l \in \{t,f\}} v_{ml}^{j} r_{ml} \sum_{A \in F_k} \prod_{a \in A} p_a \prod_{b \in A^C} (1 - p_b) \tag{4}$$

where $\Pr(K = k) = \sum_{A \in F_k} \prod_{a \in A} p_a \prod_{b \in A^C} (1 - p_b)$ is the Poisson Binomial distribution describing the probability that k receivers engage, where F_k encodes the possible subsets of k integers that can be selected from N, i.e. the possible combinations of receivers who can engage with a piece of news to produce overall engagement level k. The set A is just a particular such subset while A^C is its complement. The receiver strategy is just the probability p_a that a given receiver a engages. These probabilities may be different for different receivers and may depend on the full history of play of the receivers and transmitter i.e. we place no particular constraint on the receiver strategy used. The master equation for v_{kf} is defined in the equivalent way with $(1 - r_{ml})$ in place of r_{ml} in Eq. 4.

At equilibrium the solution to Eq. 4 must satisfy $v_{ki}^{j+1} = v_{ki}^{j} = v_{ki}$. As stated above, our goal is show that a transmitter strategy $\mathbf{r}$ of the form Eq. 1 generates an equilibrium solution satisfying Eq. 3 for a single transmitter and receiver. Note that we assume that receiver and transmitter actions are subject to a small execution error at rate ε, which ensures that the Markov process described by Eq. 4 has no absorbing states, and a unique stationary distribution. And so, since we assume an infinitely repeated game, the dynamics are guaranteed to converge to this equilibrium.

1.3 Stationary distribution

The stationary distribution for the infinitely repeated game is encoded by the vector of probabilities $\mathbf{v}$. Each entry $v_{ki} \in [0, 1]$ describes the stationary probability that the game is in state (k, i), where $k \in \{0, 1, 2, \ldots N\}$ gives the number of receivers who choose to engage with the piece of news shared by the transmitted, and $i \in \{t, f\}$ encodes the type of news shared. The master equation for the Markov process associated with the infinitely repeated game is given by Eq. 4. At equilibrium, for a true news item $i = t$, this equation has the form

$$v_{kt} = \sum_{m=0}^{N} \sum_{l \in \{t,f\}} v_{ml} r_{ml} \sum_{A \in F_k} \prod_{a \in A} p_a \prod_{b \in A^C} (1 - p_b). \tag{5}$$

An analogous equation holds for $i = f$ by replacing r_{ml} with $(1 - r_{ml})$.

In order to solve for the stationary distribution of this process, marginalized over all possible overall engagement values k, we first note that the overall probability of sharing misinformation by the transmitter is

$$v_f = \sum_{k=0}^{N} v_{kf} \tag{6}$$

and $v_t = 1 - v_f$ is the overall probability of production of true news by the transmitter. As Eq. 6 emphasises, the quantities v_{kf} are marginal quantities for the stationary distribution of the iterated game.

Applying this definition to Eq. 5 we recover

$$v_t = \sum_{m=0}^{N} \sum_{i \in \{t,f\}} v_{mi} r_{mi} \tag{7}$$

since in summing over all possible engagement levels k, we sum over the probabilities of all possible combinations of receiver overall engagement which necessarily total 1. If we now set $r_{mt} = \alpha + \sum_l \gamma_l \left(\frac{m}{N}\right)^l$ and $r_{mf} = \beta + \sum_l \theta_l \left(\frac{m}{N}\right)^l$ in Eq. 7 we recover

$$v_t = \alpha v_t + \beta v_f + \sum_{m=0}^{N} \sum_{l} \left(v_{mt} \gamma_l \left(\frac{m}{N}\right)^l + v_{mf} \theta_l \left(\frac{m}{N}\right)^l \right). \tag{8}$$

where the terms γ_l and θ_l are polynomial coefficients describing the response function of the transmitter to receiver engagement, given they previously shared true or false stories respectively. If we define v_{tc} as the average rate with which receivers consume true stories, then

$$v_{tc} = \sum_{m=0}^{N} v_{mt} \frac{m}{N}$$

(where the average rate of consuming false stories v_{fc} is defined analogously). We can also define

$$(v_{tc})^l = \sum_{m=0}^{N} v_{mt} \left(\frac{m}{N}\right)^l \tag{9}$$

where $(v_{tc})^l$, is the lth raw moment of the distribution of transmitter consuming accurate news stories. i.e. $(v_{tc})^1 = v_{tc}$ is the average overall probability of receivers engaging with true news and so on. And so Eq. 8 can be written as

$$v_t = \alpha v_t + \beta v_f + \sum_l \gamma_l (v_{tc})^l + \theta_l (v_{fc})^l$$

and we recover an expression for the receiver engagement with true and false news in terms of the transmitter strategy. Next we note that when there is only one receiver and one transmitter, $(v_{tc})^l = v_{tc}$ and $(v_{fc})^l = v_{fc}$ for all $l > 1$ and so we can write $\gamma = \sum_l \gamma_l$ and $\theta = \sum_l \theta_l$ to give

$$v_t = \alpha v_t + \gamma v_{tc} + \beta v_f + \theta v_{fc}$$

Replacing $v_f = 1 - v_t$ and rearranging gives us the relationship between the proportion of transmitted stories that are false and the overall probability with which receivers engage with true and false stories, presented in Eq 3 of the main text, namely:

$$v_f = \frac{1-\alpha}{1-\alpha+\beta} - \frac{\theta}{1-\alpha+\beta} v_{fc} - \frac{\gamma}{1-\alpha+\beta} v_{tc}$$

Note that this expression holds for any polynomial transmitter strategy and an arbitrary receiver strategy when interactions are pairwise.

This equation, (i.e. Eq. 3 of the main text) holds when there is a single transmitter and receiver, or when there are multiple receivers and the transmitter uses only a linear response function. In the more general case of multiple transmitters and a non-linear response function, we recover a more general relationship in terms of the moments of the stationary distribution of the iterated game, i.e.

$$v_t = \alpha v_t + \beta v_f + \sum_l \gamma_l (v_{tc})^l + \sum_l \theta_l (v_{fc})^l \tag{10}$$

and so a transmitter can induce positive or negative correlations in the same way as for the simpler case of Eq. 3, by choosing a response function with positive or negative coefficients as desired.

1.4 Responsive strategies

We have shown that transmitter strategies described by Eq 1 produce equilibrium game dynamics that satisfy Eq 3. In the remainder of this section we explore the consequences of this relationship for patterns of news consumption by receivers. To do this we consider the relationship between the amount of fake news produced by transmitters, v_f, and the total amount of news consumed receivers, v_c for different choices of transmitter strategy. These analytical results are then used to interpret the numerical results of Section 3.

We focus on a class of transmitter strategies that we call "responsive strategies". We define a responsive strategy as a strategy which assuredly increases the amount of news served to receivers of a given type as the receivers increase their engagement, in a manner that reflects the preferences of the transmitter. The reason we consider this type of strategy is that it ensures a receiver who engages more with the output of a particular transmitter will be exposed to more news of the type preferred by the transmitter.

A responsive misinformation strategy increases the probability of sharing misinformation stories as receivers increase their engagement. For this to be true in the case of a single receiver (or in the case of multiple receivers and a linear transmitter strategy), the coefficients $\frac{\theta}{1-\alpha+\beta}$ and $\frac{\gamma}{1-\alpha+\beta}$ in Eq. 3 must be negative. The more general case of a non-linear transmitter strategy and multiple

receivers is discussed in Section 1.6 below.

In order for a strategy to be viable Eq.1 must produce viable probabilities, i.e. $r_{ki} \in [0, 1]$. For a single receiver (or a linear strategy and multiple receivers), in order for a strategy to be viable requires $0 \leq \alpha < 1$, $0 \leq \beta < 1$, which implies that $1 - \alpha + \beta > 0$. Thus a responsive misinformation strategy requires $\theta < 0$ and $\gamma < 0$. In order for the choices of θ and γ to produce a viable strategy requires $-\alpha \leq \gamma \leq 1 - \alpha$ and $-\beta \leq \theta \leq 1 - \beta$.

And so a viable responsive misinformation strategy requires $-\alpha \leq \gamma < 0$ and $-\beta \leq \theta < 0$. Equivalently, a responsive mainstream news (i.e. accurate news spreading) strategy requires $0 < \gamma \leq 1 - \alpha$ and $0 < \theta \leq 1 - \beta$.

We have defined a responsive transmitter strategy as one that always serves more news of their preferred type to receivers who consume more news. Thus a responsive misinformation strategy serves more fake stories to receivers who engage more with the transmitter's stories. In contrast, receivers who engage little with the transmitter's stories are targeted with more true stories and thus are incentivized to engage more. As we show through simulation in the main text, and in Section 3 below, a transmitter who uses a responsive misinformation strategy against truth-seeking receivers, who try to increase their benefit from true stories by engaging more, produces game dynamics in which receivers engage more with misinformation than with accurate stories on average. The conditions for a transmitter strategy to be responsive are simply $0 < \gamma \leq 1 - \alpha$ and $0 < \theta \leq 1 - \beta$.

Iterated game strategies that enforce different types of correlation have been studied extensively in the context of the iterated prisoners dilemma [11, 14, 13, 8], where it has been shown that one player can "extort" their opponent, particularly if the opponent engages in a process of noisy optimization. We define an extortion strategy for the misinformation games as a responsive strategy that additionally enforces $v_f > v_c$ (for misinformation sites), where $v_c = v_{tc} + v_{fc}$ is the overall probability of engagement by receivers. Similarly, for mainstream sites extortion strategies must enforce $v_t > v_c$. The extortion strategies studied here ensure that, as the receiver optimizes their strategy, the rate of increase in fake (or true) news exceeds the rate of increase of overall engagement by receivers. In the next section we provide conditions for a transmitter strategy to be an extortion strategy. In Section 3 we show by simulation that a large proportion of successful transmitter

strategies are extortion strategies.

1.5 Necessary and sufficient conditions for extortion under linear transmitter strategies

The definition of a extortionate misinformation site is that $v_f > v_c$ for all receiver strategies. In this section we provide conditions for a transmitter strategy to satisfy this condition in the case of a single receiver (or in the case of multiple receivers and a linear transmitter strategy).

In order to find necessary and sufficient conditions for a transmitter strategy to enforce such a relationship, first define the following parameters

$$\kappa = \frac{1-\alpha-(\gamma-\theta)/2}{1-\alpha+\beta+\theta}$$

$$\lambda = -\frac{(\gamma-\theta)/2}{1-\alpha+\beta+\theta}$$

$$\chi = -\frac{\gamma+\theta}{2(1-\alpha+\beta-(\gamma-\theta)/2)} \tag{11}$$

This choice of transform comes from previous work on iterated games [14, 1] and we do not discuss the approach in detail here, but simply reference the relevant literature.

Note that $|\chi| \leq 1$ is required to produce a viable strategy. Note also that the denominator in Eq 11, namely $(1-\alpha+\beta-(\gamma-\theta)/2)$, is required to be positive to produce a viable strategy and thus any misinformation promoting responsive strategy necessarily has $\chi > 0$.

Converting our expression Eq. 3 into the new parameters (i.e substituting Eq. 11 into Eq. 3) produces the following expression

$$v_f - \kappa + \lambda(v_{tn} + v_{fc}) = \chi\left[v_c - \kappa + \lambda(v_{tn} + v_{fc})\right] \tag{12}$$

where v_{tn} is the probability with which true news is shared but not consumed, i.e. $v_{tn} = v_t - v_{tc}$.

Eq. 12 is convenient because it allows us to state the conditions for extortion as i) $\chi > 0$ (which is necessary for the strategy to be responsive) and ii) $v_c \leq \kappa + \lambda(v_{tn} + v_{fc})$ for all possible receiver strategies (which is necessary to ensure $v_f > v_c$, given that $|\chi| \leq 1$). If these two conditions are met, the strategy is responsive and $v_f > v_c$ for all receiver strategies, and so the strategy is an extortioner.

The first condition is met provided $\gamma + \theta < 0$ (since $\gamma < 1 - \alpha$ is required to produce a viable strategy). To asses the second condition note that ii) implies $v_f + v_c \leq 2\kappa - 2\lambda(v_{tn} + v_{fc})$. Also note that $v_f + v_c = 1 + v_{fc} - v_{tn}$ and so condition ii) is equivalent to

$$1 + v_{fc} - v_{tn} \leq 2\kappa - 2\lambda(v_{tn} + v_{fc}) \tag{13}$$

All that remains is to inspect the limiting cases, where this conditions is most stringent. A receiver who never consumes enforces $v_{fc} = 0$ and Eq. 13 becomes $1 - v_{tn} \leq 2\kappa - 2\lambda v_{tn}$ which is guaranteed to hold provided

$$\kappa \geq 1/2$$

and

$$\kappa \geq \lambda \tag{14a}$$

The other limiting case of a receiver who always consumes enforces $v_{tn} = 0$ and generates conditions

$$\kappa \geq 1/2$$

and

$$\kappa \geq 1 + \lambda. \tag{14b}$$

Eq. 14b is the more stringent of these two conditions and thus it provides necessary and

sufficient conditions for extortion. Substituting back from Eq. 11 these conditions become

$$\theta = -\beta$$

and

$$\alpha + \beta + \gamma \leq 1. \tag{15}$$

The equivalent condition for a mainstream transmitter is

$$\gamma = 1 - \alpha$$

and

$$\alpha + \beta + \theta \leq 1. \tag{16}$$

And so we can define an extortionate misinformation strategy [11] as one that ensures the amount of misinformation shared always exceeds the amount of news consumed i.e. $v_f > v_{fc} + v_{tc}$. For a misinformation transmitter using a linear strategy, this has the form $\mathbf{r}^*_- = \{\theta = -\beta, 1 - \alpha - \beta < \gamma \leq 0\}$ (see below). Conversely, an extortion strategy for a transmitter seeking to spread accurate information ensures that the amount of true news shared always exceeds the amount of news consumed i.e. $v_t > v_{fc} + v_{tc}$. For such a transmitter a linear extortion strategy has the form $\mathbf{r}^*_+ = \{\gamma = 1 - \alpha, 0 \leq \theta < 1 - \alpha - \beta\}$.

1.6 Nash equilibria in the misinformation game

In our model we typically assume that inattentive receivers who noisily optimize their engagement strategies seek to consume true stories. Such a process does not necessarily lead to optimal receiver behavior, or even to behaviors predicted by standard solution concepts such as Nash equilibria. However, it is useful to supplement our results by characterizing the Nash equilibria for the misinformation game. In order to perform this analysis we set the receiver payoffs as $\pi_f = -C$ and $\pi_t = B$, where $C > 0$ is the cost of consuming misinformation and $B > 0$ is the benefit of consuming

accurate information.

For an accurate news transmitter, which seeks to promote accurate information, there is no conflict between the incentives of transmitter and receiver, and so they both should adopt a Nash equilibrium in which the receiver always engages and the transmitter only shares true stories. For a misinformation transmitter, which seeks to promote false news, there is a conflict between the incentives of transmitter and receiver.

The Nash equilibria for the two-player misinformation game can be easily characterized using the Folk Theorem for infinitely iterated games [6]. The minimax payoff for receivers in the misinformation game is 0, which is achieved by never consuming. If the receiver does consume, the transmitter can always hold them to a lower payoff by transmitting misinformation. The minimax payoff for the transmitter is also 0, provided we assume the payoff to the transmitter of misinformation being consumed is $b_f \geq 0$ and also the payoff from true news being consumed is $b_t \geq 0$, whereas the payoff when no news is consumed is 0, and so the transmitter's minimax profile is uniquely determined by a receiver strategy that never consumes (Table S1). Thus any feasible payoff profile that provides a positive payoff to both players is individually rational.

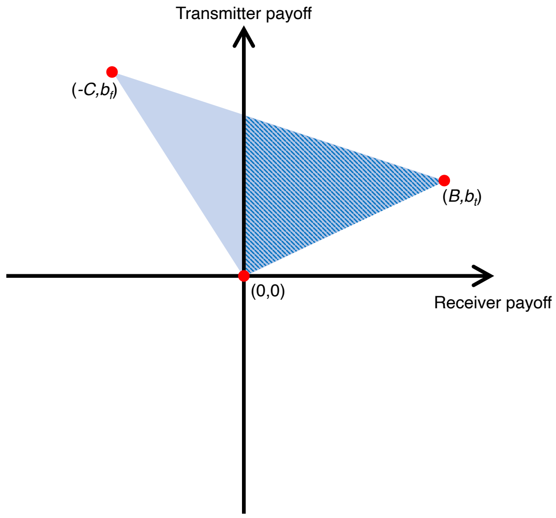

Figure S1: **Feasible, individually rational payoffs for the misinformation game** – The set of feasible payoffs (blue region) and the subset of individually rational payoffs for both players (striped region) for a misinformation game with $b_f > b_t$ meaning that the transmitter prefers to generate engagement with misinformation. Individually rational payoffs for both players only arise when both players receive a positive payoff. An equilibrium in which the receiver always consumes and the transmitter always shares misinformation, $(-C, b_f)$ is feasible but not individually rational.

Any individually rational, feasible payoff is a Nash equilibrium of the game (Figure S1), and, since the game is assumed infinite, can always be enforced by players employing a trigger strategy. That is, if the receiver deviates from the prescribed course of play, the transmitter switches to only sharing misinformation. Any benefit gained by the receiver from deviating is lost in the long run, since the best they can do is to cease consuming and receive zero payoff in every subsequent round. Conversely, if the transmitter deviates from the equilibrium, the receiver can switch to never consuming news, with the same result that any benefit to the transmitter gained by deviating eventually lost. As discussed above, the set of individually rational, feasible payoffs (Figure S1) in the misinformation game corresponds to cases in which both transmitter and receiver have positive payoffs. In the case where a misinformation site receives payoffs $b_t = 0$ and $b_f > 0$, this nonetheless means that they must share a mixture of both true and misinformation, since the payoff that results from only sharing misinformation produces a negative payoff to the receiver.

To explore this point further, consider a misinformation site who simply transmits true stories with fixed probability r each round. The payoff to a receiver who consumes news from the receiver at equilibrium probability v_c is simply $w_t = v_c(Br - C(1-r))$, which is positive provided $r > \frac{C}{B+C}$. If this condition is met, the receiver's payoff always increases with overall engagement, and so an equilibrium at which the receiver always consumes and the transmitter shares true stories with probability $r > \frac{C}{B+C}$ can be enforced as a Nash equilibrium of the misinformation game (e.g. by employing a trigger strategy, as discussed above). More generally, this condition is important because it shows that, if consumers don't dislike misinformation enough, news sites can successfully generate engagement with false stories without any need to be responsive. This also illustrates the need, in a general sense, for a misinformation site to "mash up" true and fake stories to produce engagement. Finally, note that if $b_t > 0$, $r = 1$ and $v_c = 1$ produces positive payoffs for both players, and can also be enforced as a Nash equilibrium – i.e. mainstream news sites do not need to mash up true and fake stories when faced with rational receivers who value true news.

Thus our model predicts that misinformation sites should always share a mixture of true and fake stories if their readers value accuracy, regardless of whether those readers are rational (as per this Nash equilibrium calculation), or lazy and irrational (as per our simulations above).

1.7 Modelling local optimization

For the most part we do not assume that receivers in the misinformation game are perfectly rational, but rather update their behavior according to a local optimization process [15]. Under this process a receiver temporarily adopts an alternate strategy b to the one they are currently employing, a, and compares the payoff they receive with the new strategy to that received under the old strategy. They then adopt the new strategy, and discard the old, with probability $\pi_{a\to b}$ determined by a Fermi function.

$$\pi_{a\to b} = \frac{1}{1+\exp[\sigma(w_a - w_b)]} \tag{17}$$

where w_i is the payoff received under strategy a, and σ determines the level of attention the player pays to their payoffs. If $\sigma < 1$ little attention is paid to payoffs and the optimization process is very noisy, with receivers frequently adopting suboptimal engagement strategies. If $\sigma \gg 1$ a high level of attention is paid to payoffs, and only strategies that improve payoffs are adopted.

When choosing the alternate strategy we assume that b is small perturbation $\Delta_\mu \in [0, 0.05]$ to the current strategy, such that each element of the receiver strategy $\mathbf{p} = \{p_0, p_{ct}, p_{cf}, p_{nt}, p_{nf}\}$ is increased or decrease by an independently drawn Δ_μ, with the constraint that each element must remain a viable probability (i.e. $p_{ij} \in [0, 1]$). See [12] for an overview of possible update rules.

1.8 Time series for linear and non-linear transmitter strategies

In the main text we present the time series for co-optimization (Figure 2) for a transmitter strategy that employs non-linear feedback, defined by $r_{kt} = 1/(1+\exp[\lambda(k/N - 0.5)])$ and $r_{kf} = 1/(1+\exp[\lambda(k/N - 0.25)])$, where we set $\lambda = 100$ and the population of receivers to be $N = 100$. In this example the non-linear feedback can be expressed as a Taylor series as

$$r_{kt} = 1 + \sum_{n=0}^{\infty} \frac{(-1)^{n+1}\lambda^n E_n(0)}{2n!} \left(\frac{k}{N} - \frac{1}{2}\right)^n$$

similarly

$$r_{kf} = 1 + \sum_{n=0}^{\infty} \frac{(-1)^{n+1}\lambda^n E_n(0)}{2n!} \left(\frac{k}{N} - \frac{1}{4}\right)^n$$

where $E_n(0)$ is the nth Euler polynomial evaluated at 0. This allows us to compute the coefficients γ_i and θ_i in Eq. 10. In general this must be done numerically. Here we have $\alpha = \beta = 1/2$ and

$$\gamma_1 = \sum_{n=1}^{\infty} \frac{(-1)^{n+1}\lambda^n E_n(0)}{2n!} \left(\frac{1}{2}\right)^{n-1} = \frac{1 - \exp[\lambda/2]}{1 + \exp[\lambda/2]}$$

and

$$\theta_1 = \sum_{n=1}^{\infty} \frac{(-1)^{n+1}\lambda^n E_n(0)}{2n!} \left(\frac{1}{4}\right)^{n-1} = 2 \times \frac{1 - \exp[\lambda/4]}{1 + \exp[\lambda/4]}$$

and so for this strategy the first-order feedback terms satisfy $\gamma_1 < 0$ and $\theta_1 < 0$, i.e. they tend to generate a negative correlation between misinformation and engagement. This is illustrated in the time-series in Figure 2 shows how a transmitter using this strategy induces engagement with misinformation in a population of optimizing transmitters.

The same effect can be produced for simple linear strategies, as shown in Figure S2 below. Here a transmitter using a fixed linear strategy $\mathbf{r} = \{1.0, 0.1, -0.9, -0.1\}$ interacts with an optimizing receiver. While the time-series produced do not show a visibly obvious correlation, when we plot the rate of misinformation production against the rate of engagement, we see an unambiguous correlation, as predicted by Eq. 3, and analogous to the effects shown for the nonlinear transmitter strategy in Figure 2.

1.9 Modelling optimization through social learning

In addition to local optimization, we also model receivers whose strategies are updated through a process of cultural evolution via payoff-biased imitation [15]. Under this model, a pair of receivers, a and b, are selected at random from the population of receivers. Receiver a then adopts the strategy of receiver b with probability

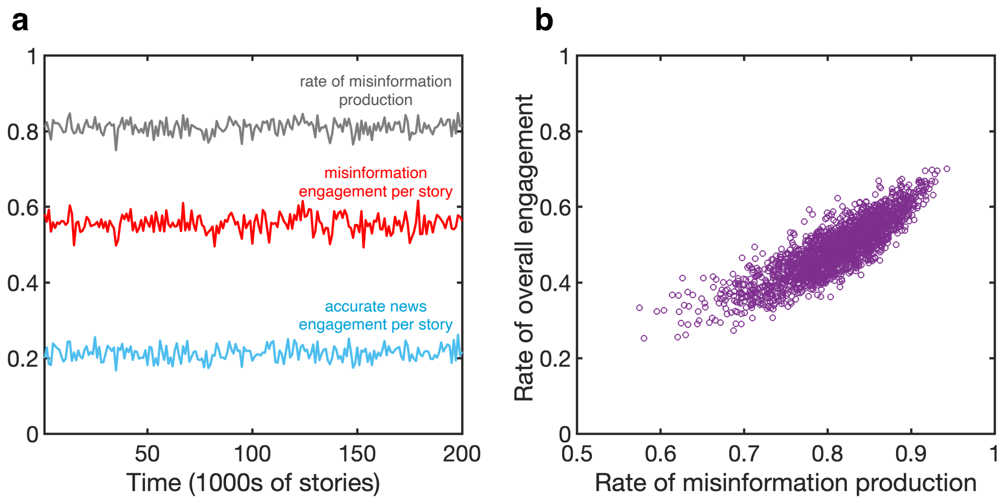

Figure S2: **Linear transmitter driven engagement with misinformation.** An illustrative example of transmitter-driven engagement with misinformation for a linear transmitter strategy. We selected a transmitter strategy which employs linear feedback, $\mathbf{r} = \{1.0, 0.1, -0.9, -0.1\}$, which induces a positive correlation between rate of misinformation production and engagement (Eq. 3). a) Dynamics of engagement with accurate news (blue), misinformation (red) by the receiver, and overall production of misinformation (grey) by the transmitter, for a receiver employing a noisy optimization process with low attention ($a_0 = a_1 = 0$ and $\sigma = 1$). b) The result of these dynamics are a positive correlation between engagement rate per article and rate of misinformation production. In all cases receiver mutations were local (see SI section 1.5) and we set receiver payoffs $\pi_t = 2$ and $\pi_f = -1$.

$$\phi_{a \to b} = \frac{1}{1 + \exp[\sigma(w_a - w_b)]} \tag{18}$$

where w_a is the expected payoff to player a at equilibrium in the infinitely repeated game. In addition, mutations are introduced to the receiver population at rate μ, under which a player spontaneously adopts a news strategy drawn uniformly from the space of possible receiver strategies. Note that Eq. 17 and Eq. 18 are identical, but here b is drawn from the strategies present in the population, while in Eq. 17, b is chosen as a perturbation to the current strategy. As such, the process of copying other players' strategies tends to result in many different players using the same strategy, compared to the case where each player independently optimizes their own strategy.

1.10 Feedback and receiver engagement

Finally, we show that in order to produce the region F2 or T2 of main text Figure 3, feedback is required in the strategies used by transmitters. To see this, consider a transmitter strategy without feedback, such that $r_{mt} = r_{mf} = \alpha$, i.e. misinformation is produced at a constant rate $1 - \alpha$. The probability of engagement with misinformation by an arbitrary (but inattentive, $a_0 = 0$)) receiver strategy $\mathbf{p}$ is then

$$\begin{aligned} v_{ct}^{j+1} &= \alpha(v_{ct}^{j} p_{ct,H} + v_{nt}^{j} p_{nt,H} + v_{cf}^{j} p_{cf,H} + v_{nf}^{j} p_{nf,H}) \\ v_{cf}^{j+1} &= (1-\alpha)(v_{ct}^{j} p_{ct,H} + v_{nt}^{j} p_{nt,H} + v_{cf}^{j} p_{cf,H} + v_{nf}^{j} p_{nf,H}) \end{aligned}$$

where j is the round of play, and $p_{ab,H}$ is the probability of engaging given that the previous round had outcome ab and H is the full history of interaction by the receiver with the transmitter. Under this model

$$v_t = \alpha$$

and so at equilibrium we have a difference in engagement probability by the receiver of

$$\frac{v_{ct}}{v_t} - \frac{v_{ft}}{v_f} = \frac{1}{\alpha} v_{ct} - \frac{1}{1-\alpha} v_{cf} = 0$$

and so no difference in engagement is produced when transmitter strategies do not use feedback, provided receiver strategies are inattentive (i.e. are not making use of prior knowledge of veracity when deciding to engage). If, in contrast, receivers are attentive and see true news we have

$$\begin{aligned} v_{ct}^{j+1} &= (1-a_0)\alpha(v_{ct}^{j} p_{ct,H} + v_{nt}^{j} p_{nt,H} + v_{cf}^{j} p_{cf,H} + v_{nf}^{j} p_{nf,H}) + a_0 \\ v_{cf}^{j+1} &= (1-a_0)(1-\alpha)(v_{ct}^{j} p_{ct,H} + v_{nt}^{j} p_{nt,H} + v_{cf}^{j} p_{cf,H} + v_{nf}^{j} p_{nf,H}) \end{aligned}$$

and the engagement difference at equilibrium is

$$\frac{v_{ct}}{v_t} - \frac{v_{ft}}{v_f} = \frac{1}{\alpha}v_{ct} - \frac{1}{1-\alpha}v_{cf} = a_0/\alpha$$

i.e. the engagement difference reflects the preferences of the receiver. Since region F2 and T2 represent scenarios in which engagement patterns are at odds with receiver preference, we see that absent feedback, region F2 and region T2 cannot be produced.

2 Comparison to previous models

In this section we compare our model to three previously proposed theories of misinformation engagement and/or production [2, 16, 9]. We ask whether the theories presented in these papers can produce region F2 and T2 of main text Figure 3, and thereby explain our empirical results (main text Figure 4). That is, we ask whether our proposed theory is uniquely predictive of the observed data. We find that, indeed, the other theories cannot reproduce the observed patterns since they use transmitter strategies that do not employ feedback (see Section 1.7 above).

2.1 Vosoughi et. al. 2018

Vosoughi et. al. [16] present empirical results showing that, among news stories that were questionable enough to get fact-checked – by snopes.com, politifact.com, factcheck.org, truthor-fiction.com, hoax-slayer.com, or urbanlegends.about.com – false news spread further on social networks and persisted longer. They also find that false news was more novel, in an information-theoretic sense, than true news. The authors are careful to note that "Although we cannot claim that novelty causes retweets or that novelty is the only reason why false news is retweeted more often, we do find that false news is more novel and that novel information is more likely to be retweeted."

This suggests that readers derive benefit from engaging with misinformation due to its novelty. However such an explanation, absent feedback between receiver and transmitter sharing strategy, would predict greater engagement with false stories among both fake and mainstream sites , which is not consistent with our empirical findings (main text Figure 4).

2.2 Pennycook et. al 2021

Pennycook et. al [9] investigate the role of inattention in shaping consumer engagement with misinformation. The authors present a model in which receiver utility is derived from a consumer's preferences and the level of attention they pay to a topic. Fitting the model parameters to experimental data they find the best-fit preference parameters indicate that participants value accuracy as much as or more than partisanship. Thus, they would be unlikely to share false but politically concordant content if they were attending to accuracy and partisanship" suggesting a model in

which consumers prefer true news to false news, and occasionally share misinformation accidentally, due to inattention. This account would therefore predict greater engagement with true stories than false stories, regardless of whether the producer is a misinformation or mainstream site , which is inconsistent with our empirical findings (main text Figure 4).

2.3 Allcott and Gentzkow 2017

Allcott and Gentzkow [2] develop a model of supply and demand of misinformation. Under this model "misinformation arises in equilibrium because it is cheaper to provide than precise signals, because consumers cannot costlessly infer accuracy, and because consumers may enjoy partisan news."

This model considers consumer decisions at the level of the news outlet (e.g. "shall I subscribe to this newspaper or not?") rather than at the level of the individual story, and thus does not make direct predictions about our key empirical outcome, the relative probability of engagement for true versus false news within a given outlet. However, the model's implications can be extrapolated to our setting. On the demand side, this means that consumers/receivers either (i) engage with misinformation accidentally due to inattention or (ii) derive benefit from engaging with misinformation due to e.g. partisan preference. Inattention alone, absent feedback between transmitter and receiver sharing strategy, leads in the limit to equal levels of engagement with true and false news. Similar to the case of a consumer preference for novelty discussed above, a partisan preference for false news, absent feedback between receiver and transmitter sharing strategy, would predict greater engagement with false stories among both fake and mainstream sites, which is not consistent with our empirical findings (main text Figure 4).

On the supply side, this theory posits that transmitters broadcast false news because it is cheaper to produce than true news (i.e. the net payoff for transmitters from receivers engaging with false news is greater than it is for true news). By this logic, it is payoff maximizing for transmitters to produce false news so long as the engagement advantage of true news over false news is sufficiently small (or non-existent) – i.e. due to receivers being inattentive and/or preference for falsehoods. This explanation for the production of misinformation, while entirely plausible, makes

no prediction for the different patterns of engagement among consumers with mainstream versus misinformation sources, which is our key empirical result (main text Figure 4).

3 Further analysis and extensions of the model

In this section we use simulations to perform robustness checks on our model, and show that our results hold as we relax our assumptions and alter our model specification. We show that i) our results are robust to different definitions of "successful" transmitters ii) our results are robust to different models of receiver learning, iii) our results are robust to different receiver group sizes but require a moderate to high degree of transmitter microtargetting, iv) our results require low levels of receiver attention, v) our results are robust to competition between transmitters and vi) our results are robust to changes to consumer demand for misinformation.

3.1 Identifying successful transmitter strategies

In order to find successful mainstream and misinformation transmission strategies we generated 10^8 receiver strategies as described in main text Materials and Methods. We then defined successful misinformation transmitters as those which achieved i) in the 90th percentile for misinformation engagement, and ii) a higher probability of transmitting fake than true news, $v_f > 0.5$. Similarly, we defined successful mainstream transmitters as those in the 90th percentile for true news engagement who transmit a higher probability of true news than fake, $v_t > 0.5$. These definitions were used to select the strategies used to produce Figure S3 below and discussed in the main text.

The most successful mainstream and misinformation dissemination strategies induce characteristic, and opposite, patterns of engagement among readers in our model (Figure 3). In particular, successful misinformation sites induce higher reader engagement with each false news story, as well as greater overall engagement of false news than true news (Table S2). This phenomenon arises even though we assume that receivers strictly prefer true news over false news, so there is no inherent appeal of false news stories under our model.

To understand this phenomenon we inspected the strategies of successful misinformation sites under our model. We find that 100% of misinformation sites indeed use responsive strategies that enforce a positive correlation between engagement and false news transmission. A significant proportion (72%, see SI Section 1) use a type of extortion strategy [11] that enforces $v_f \geq v_{fc} + v_{tc}$, i.e. successful misinformation site strategies tend to increase their false news output rapidly in

response to increased engagement by a given user. However, if engagement drops, they tend to increase their output of true stories (to draw the user back in). As a result, they "mash up" true and fake stories as engagement fluctuates over time.

The behavior of successful mainstream sites, that seek to generate engagement with true stories, shows the opposite pattern from of successful misinformation sites. 100% of successful mainstream strategies enforce a *negative* correlation between engagement and false news transmission.

A significant proportion (56%, see SI Section 1) use a strategy that enforces $1 - v_f \geq v_{fc} + v_{tc}$, i.e. successful mainstream site strategies tend to decrease their output of false stories rapidly in response to increased engagement, but may share more false stories when engagement is low (in a misguided attempt to draw readers back in).

The full, unfiltered distribution of engagement per story and overall engagement for the generated strategies is shown in Figure S4a-b. The distributions have a weak bias towards true news consumption ($p < 0.001$) reflecting the noisy optimization process of the receivers, who prefer to consume true and avoid misinformation.

We also explored the effect of changing the definition of "successful" transmitters of true and misinformation. We look at sites in the top 10% of engagement, who share their preferred news type greater than 50% of the time. In Figure S4c-d we vary this threshold and look at the average difference between engagement per story and overall engagement. We see that engagement per story difference is insensitive to the threshold choice, whereas overall engagement difference reverses direction when the threshold is low, indicating that there are many news sites who are good at producing engagement with true or misinformation while also sharing such stories rarely.

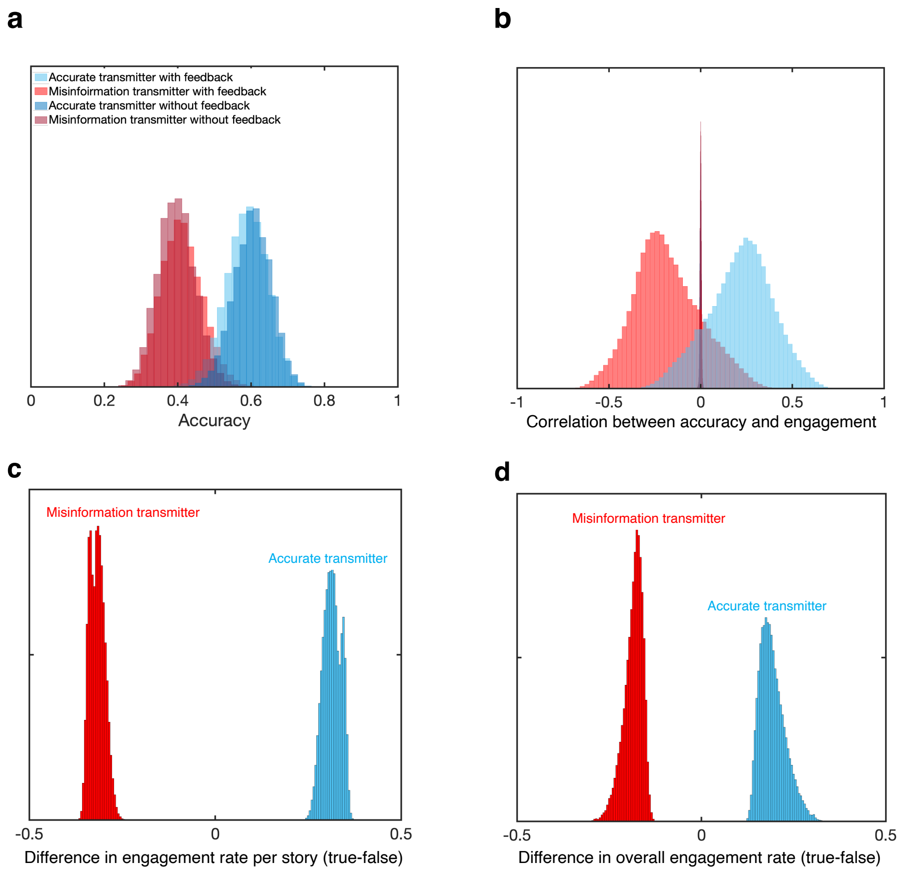

Figure S3: **Feedback dramatically changes predicted patterns of news engagement** – a) We identified strategies that are successful at promoting engagement with true news stories (accurate sites) or with misinformation stories (misinformation sites). To do this we randomly drew 10^8 transmitter strategies (Eq. 2) and allowed a single receiver, incentivized to engage with true news ($\pi_t = 2$ and $\pi_f = -1$), to optimize their engagement strategy (see SI Section 1) over the course of 10^4 interactions. The receiver was assumed to be inattentive ($a_0 = a_1 = 0$ and $\sigma = 1$). We identified the transmitter strategies that successfully promote engagement with true news stories (mainstream sites, blue), i.e those that produce a true news engagement probability within the 90th percentile of the 10^8 transmitter strategies considered, as well as $v_t > 0.5$. Similarly, we identified the transmitter strategies that successfully promote engagement with misinformation (misinformation sites, red), i.e those that produce a misinformation engagement probability within the 90th percentile, as well as $v_f > 0.5$. We plot the distribution of accuracy among the most successful transmitters, for strategies that make use of feedback ($\gamma \neq 0$ and $\theta \neq 0$, light colors) and strategies that do not make use of feedback ($\gamma = \theta = 0$). We see in both cases that accurate transmitters tend to share accurate stories and misinformation transmitters tend to share fake stories. b) We calculated the regression coefficient between engagement and accuracy for each transmitter strategy, for a group of 10^3 receivers engaging with 20 stories from each source. In the absence of feedback (dark colors) there is no correlation between accuracy and engagement, whereas in the presence of feedback (light colors) accurate transmitters generate a positive correlation while misinformation transmitters tend to generate a negative correlation. In all cases receiver strategic exploration was local (see SI section 1.5) and we assumed transmitter error rates of 0.3 (see SI Section 1). Results for other parameter choices are shown in the SI Section 3. c) We plot the difference between engagement with true and fake stories, $v_{tc}/v_t - v_{fc}/v_f$, for all mainstream and misinformation sites for strategies that use feedback

. Mainstream site strategies induce engagement with accurate stories while misinformation site strategies induce the opposite effect. d) We also report the difference in overall engagement with true stories, v_{tc}, and overall engagement with fake stories v_{fc} for strategies that use feedback. In all cases receiver mutations were local (see SI section 1.5) and we set receiver payoffs $\pi_t = 2$ and $\pi_f = -1$.

	Accurate transmitters	Misinformation transmitters
Engagement rate per story (true)	0.67 (0.65-0.69)	0.33 (0.3-0.36)
Engagement rate per story (false)	0.35 (0.32-0.38)	0.65 (0.64-0.66)
Overall engagement rate (all)	0.52 (0.51-0.53)	0.5 (0.49-0.51)
Overall engagement rate (true)	0.36 (0.33-0.39)	0.16 (0.13-0.18)
Overall engagement rate (false)	0.16 (0.13-0.19)	0.34 (0.32-0.37)

Table S2: **News engagement patterns.** Summary of the engagement rates per story and overall engagement rates for mainstream and misinformation site transmission strategies shown in Figure S3.

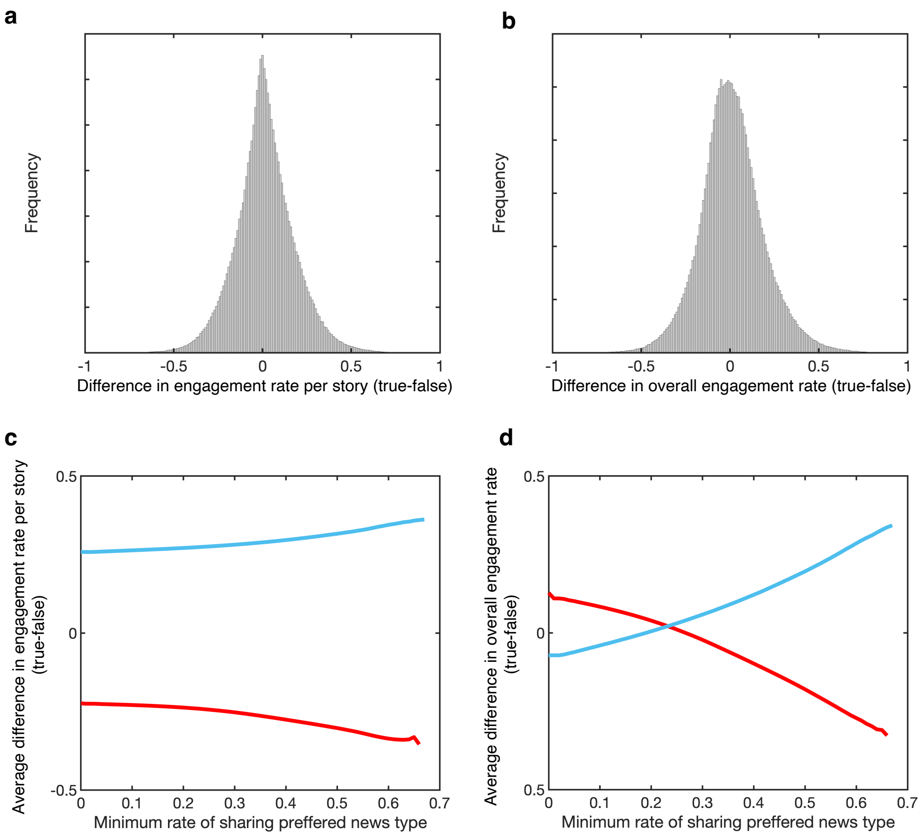

Figure S4: **Full distribution of engagement per story and overall engagement** – Shown are the distributions of the difference in engagement per story (a) and overall engagement (b) with true vs fake stories for 10^8 transmitter strategies. We define successful misinformation strategies as those with $v_f > 0.5$ and misinformation engagement, $v_{fc}/v_f \geq 0.704$ which is the 90th percentile among all observed strategies. Similarly we define successful mainstream strategies as those with $v_t > 0.5$ and misinformation engagement, $v_{tc}/v_t \geq 0.758$ which is the 90th percentile among all observed strategies. c) We calculated the average difference in engagement as a function of the threshold probability of sharing the transmitter's proffered news type used when to determine which transmitter strategies are successful. We see that both mainstream (blue) and misinformation sites (red) produce an engagement difference that is insensitive to this choice of threshold. d) However the overall engagement probability is highly sensitive to the threshold, so that when the threshold is low the preferred news type is consumed less frequently than the non-preferred type. This arises because many successful sites produce high levels of engagement with their preferred news type while sharing it rarely.

3.2 Co-optimizing transmitters and receivers

We explored the behaviors of transmitters and receivers when both players co-optimize their strategies (main text Figure 3 and Figure S5). In order to identify regions F1-F3 and T1-T3 (Figure 3) we allowed ensembles of transmitter and receiver strategies to co-optimize over 10^4 time steps (see SI Section 1). Next we then expanded this analysis to consider transmitters who seek only to maximize engagement among receivers ($b_f = b_t$), but make different assumptions about the type of news those receivers prefer. Transmitters who assume receivers prefer misinformation (e.g. because it is more novel) employ strategies that seek to reinforce increased engagement by increasing the amount of false stories they share in response (see SI Section 1). Similarly transmitters who assume receivers prefer true news employ strategies that seek to reinforce increased engagement by increasing the amount of true news they share in response. Transmitters who make no assumption about receivers preferences (and hence try out all possible strategies without bias) and transmitters who assume receivers prefer true news, both produce greater engagement with true than with false stories (Figure S5a). In contrast, transmitters who assume receivers prefer misinformation, produce greater engagement with false than with true stories.

As these results show, transmitters tend to elicit behavior among receivers that reinforce their assumptions about receiver preferences. We explored more broadly (Figure S5b) the conditions under which an assumed preference for misinformation is self-reinforcing in this way (i.e. leads receivers to engage more with false stories than with true). We find that misinformation is self-reinforcing when attention to accuracy is low (i.e. when receivers either cannot assess or do not pay attention to the accuracy of a headline) and when attention to payoffs is low. This suggests that increasing reader attention to the news they are consuming can reduce not only their own consumption of misinformation but also the production of misinformation by transmitters who make faulty assumptions about receiver preferences.

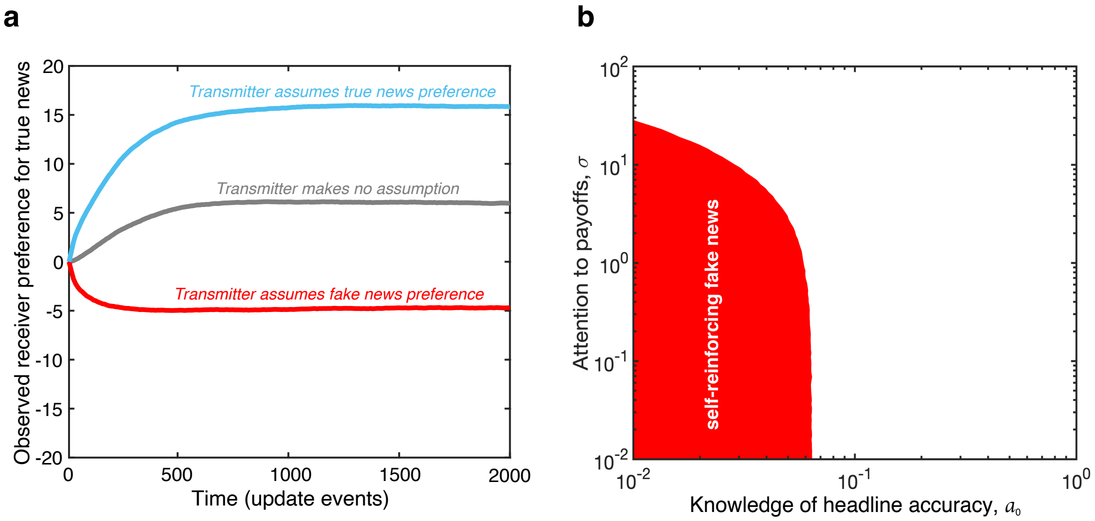

Figure S5: **Self-reinforcing misinformation** We allowed both receivers and transmitters to optimize under the process described in main text Figure 3. Here we assume that transmitters seek only engagement with news, regardless of veracity ($b_f = b_t = 1$). But transmitters draw strategies that reflect their assumptions about the receivers' preferences (see SI Section 1). a) Time series of receiver engagement as receivers and transmitters co-optimize. We initialized with a transmitter that always shares true news and a receiver that never engages. We calculated the percentage difference in probability of engagement with a true versus a fake story, averaged across 10^4 replicate simulations. When transmitters make no assumption (gray), or assume receivers prefer true news (blue), true news receives greater engagement, which reflects the underlying receiver preferences. When transmitters incorrectly assume receivers prefer misinformation (red), however, false stories receive greater engagement – that is, the assumption that receivers prefer misinformation is self-reinforcing. b) We calculated the average engagement difference for different levels of receiver attention to payoffs, σ, and for different probabilities that receivers have prior knowledge about headline accuracy, a_0. It is only when attention to accuracy and attention to payoffs are low that misinformation is self-reinforcing (red region). In all cases receiver mutations were local (see SI Section 1.5) and receiver payoffs are set to $\pi_t = 2$ and $\pi_f = -1$. Transmitter mutations were global. Receiver attention to payoffs was set at $\sigma = 1$, attention to accuracy at $a_0 = 0$ in panel a) with memory $a_1 = 0$. Transmitter attention to payoffs was set at $\sigma = 100$.

3.3 Comparison of most successful transmitters with co-evolved transmitters

In our characterization of successful misinformation transmitter strategies above we selected strategies in the 90th percentile of engagement with misinformation that also produce misinformation at a probability $v_f > 0.5$, with an analogous selection process for successful transmitters of accurate information (Figure S3). A natural alternative method for selecting transmitter strategies is to look at those that emerge via an optimization process, such as that used to produce Figure 3. Figure S6 below shows the patterns of engagement that arise for misinformation and accurate transmitter selected via this process, as compared to the top 10% of transmitters. We see that such transmitters produce the same clear pattern of engagement with receivers, i.e. misinformation transmitters generate engagement with misinformation, while accurate transmitters generate engagement with accurate stories.

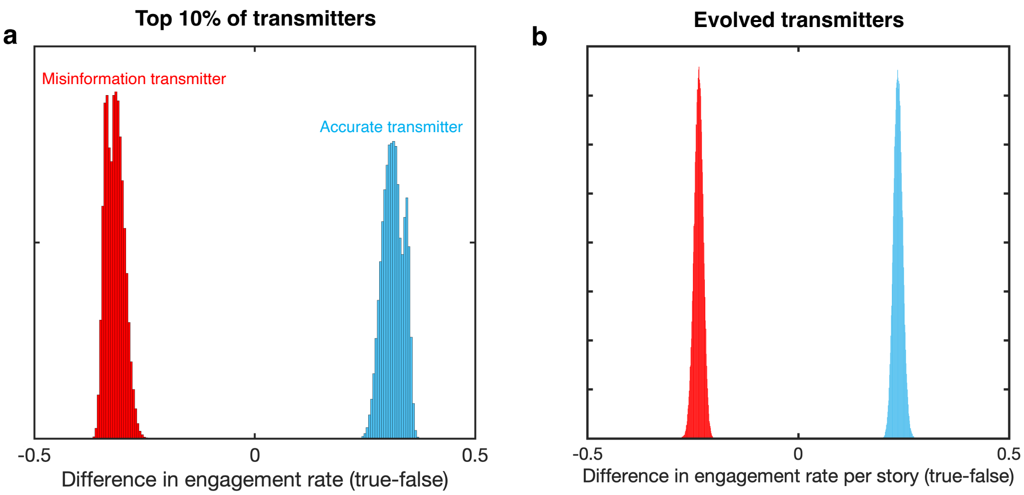

Figure S6: **Comparison of evolved transmitters to the top 10%** – a) We identified strategies that are successful at promoting engagement with true news stories (accurate sites) or with misinformation stories (misinformation sites) in the same way as in Figure S3. We plot the difference between engagement with true and fake stories, $v_{tc}/v_t - v_{fc}/v_f$, for all mainstream and misinformation sites. Mainstream site strategies induce engagement with accurate stories while misinformation site strategies induce the opposite effect. b) We plot the equivalent distribution for strategies evolved via a co-optimization process. We see a qualitatively similar pattern of engagement in both cases. In all cases receiver mutations were local (see SI section 1.5) and we set receiver payoffs $\pi_t = 2$ and $\pi_f = -1$ and transmitter payoffs $b_t = 0$ and $b_f = 1$ (for misinformation transmitters) and $b_t = 1$ and $b_f = 0$ (for accurate transmitters)

. For the optimization process, transmitter mutations were global. Receiver attention to payoffs was set at $\sigma = 1$, attention to accuracy at $a_0 = 0$ with memory $a_1 = 0$. Transmitter attention to payoffs was set at $\sigma = 100$.

3.4 Impact of absolute transmitter payoff

In our analysis and simulations we have typically assumed that transmitters who prefer misinformation receive payoffs $b_f = 1$ and $b_t = 0$, while transmitters that prefer accurate stories receive payoffs $b_f = 0$ and $b_t = 1$. This is justified since the Nash equilibria for the game are unchanged under the addition of a constant to a player's payoffs. However such a translation can impact the dynamics of an optimization process. To check the robustness of our results we reproduced Figure S6 and Figure 3 with payoffs $b_f = 2$ and $b_t = 1$ for misinformation transmitters, and payoffs $b_f = 1$ and $b_t = 2$ for accurate transmitters (Figure S7 and S8). As the figures show, both the distribution of engagement (Figure S7) and the size of the region FII in Figure 3 (Figure S8) are unchanged under these alternate payoffs.

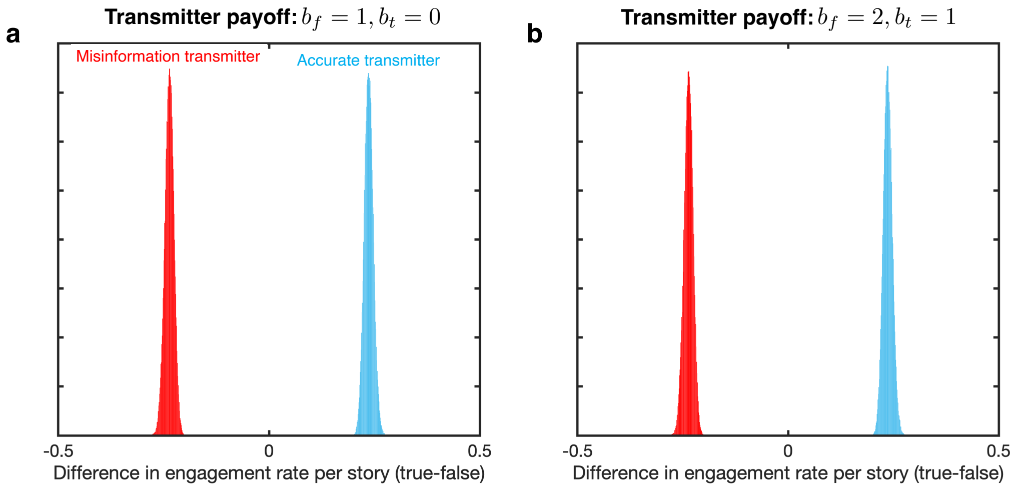

Figure S7: **Effect of transmitter payoffs on distribution of engagement under optimization** – a) We plot the distribution of engagement for strategies evolved via a co-optimization process using the same method as Figure S6, with transmitter payoffs $b_t = 0$ and $b_f = 1$. b) We compare this to the distribution of engagement for strategies evolved via a co-optimization process using the same method as Figure S6, with transmitter payoffs $b_t = 1$ and $b_f = 2$. We see no difference between the cases. In all cases receiver mutations were local (see SI section 1.5) and we set receiver payoffs $\pi_t = 2$ and $\pi_f = -1$. For the optimization process, transmitter mutations were global. Receiver attention to payoffs was set at $\sigma = 1$, attention to accuracy at $a_0 = 0$ with memory $a_1 = 0$. Transmitter attention to payoffs was set at $\sigma = 100$.

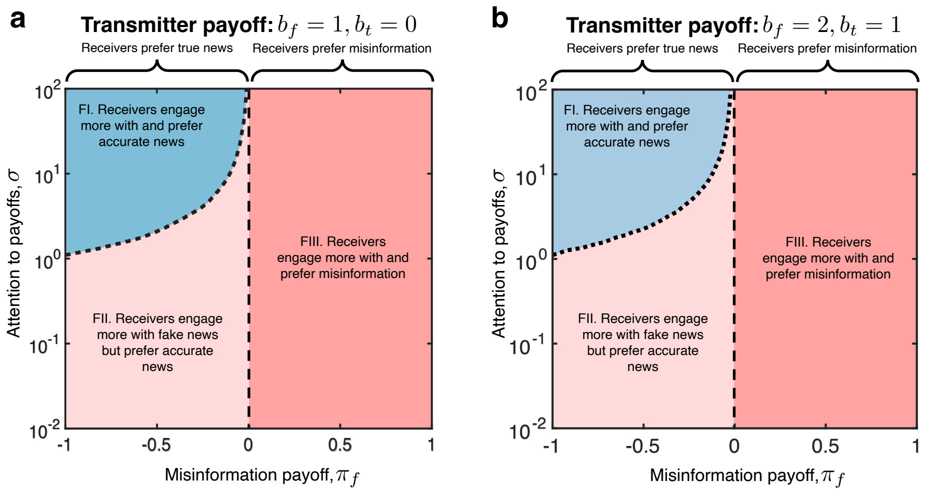

Figure S8: **Effect of transmitter payoffs, receiver preferences and attentiveness on engagement** – a) We calculated the engagement patterns among receivers optimizing under different preferences in the same way as for main text Figure 3 with transmitter payoffs set at $b_t = 0$ and $b_f = 1$. b) We compared this to a transmitter with payoffs $b_t = 1$ and $b_f = 2$. We see no difference between the patterns of engagement among receivers that emerges, as captured by the size of regions FI, FII and FIII. In all cases receiver strategic exploration was local, with $a_0 = a_1 = 0$, with transmitter attention set at $\sigma = 100$. Optimization occurred over 10^4 time-steps using ensembles of 10^3 replicates for each value of $\{\pi_f, \sigma\}$.

3.5 The effect of receiver learning model choice

We studied the effect of different receiver learning model choice. In particular we explored social learning via a model of cultural evolution, as described in Section 1.6 of this Supplement, on the level of engagement per story and overall engagement in a population of receivers. Figure S9 shows the impact of group size on engagement per story and overall engagement with fake and true news with a fixed single misinformation transmitter as in Figure S3. Assuming that the rate of strategy mutations is $\mu = 1/N$ where N is the size of the group of receivers, we see that increasing group size has no effect on overall engagement and only a modest effect on engagement per story, tending to increase true news and reduce misinformation engagement per story, without reversing the pattern of higher engagement per story with fake stories than with true.

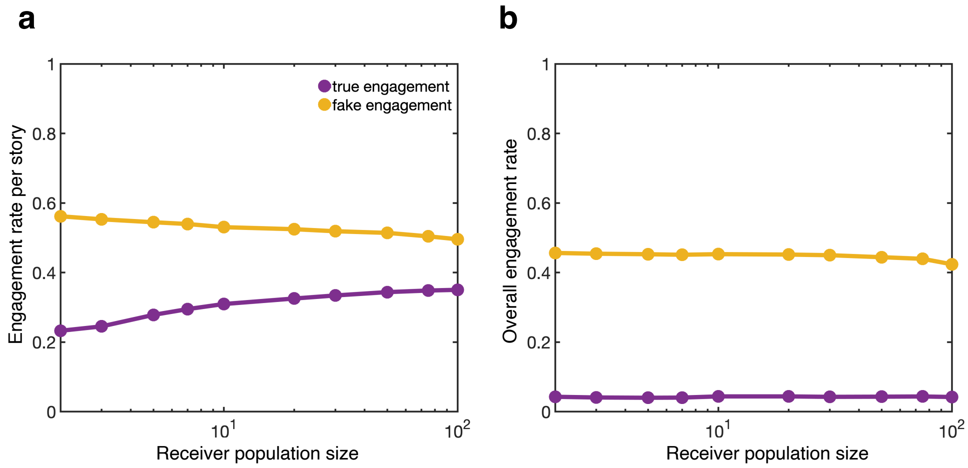

Figure S9: **Cultural evolution of receiver strategy** – a) A group of receivers evolving in response to a single transmitter, under the same process as described is Section 1.6 of the Supplement. We vary the size of the receiver group (x-axis) and calculate the equilibrium level of engagement with fake (orange) and true (purple) stories. b) We also calculate the equilibrium level of overall engagement. In all cases receiver mutations were local (see SI Section 1) and we set receiver payoffs $\pi_t = 2$ and $\pi_f = -1$. Here misinformation sites use a fixed linear responsive strategy $\mathbf{r} = \{1.0, 0.1, -0.9, -0.1\}$ see SI Section 1

3.6 The effect of receiver group size and transmitter microtargetting

We studied the effect of relaxing the assumption, employed in the main text (Figure 2-3) and in preceding sections of the SI, that a transmitter can directly target each receiver, in response to his or her engagement with the previous news article. We show that the effects observed – in particular the apparent attractiveness of misinformation – hold under a wider range of conditions in which a transmitter targets groups of individuals.

First we studied the effect of varying the size of the group of receivers engaging with a single transmitter, when all receivers use the same strategy to engage with the transmitter, with optimization taking place at the level of the group rather than at the level of the individual, as in Figure S3. Figure S10 shows that, in such a scenario, group size has no impact on either engagement per story or overall engagement even as group size varies across two orders of magnitude.

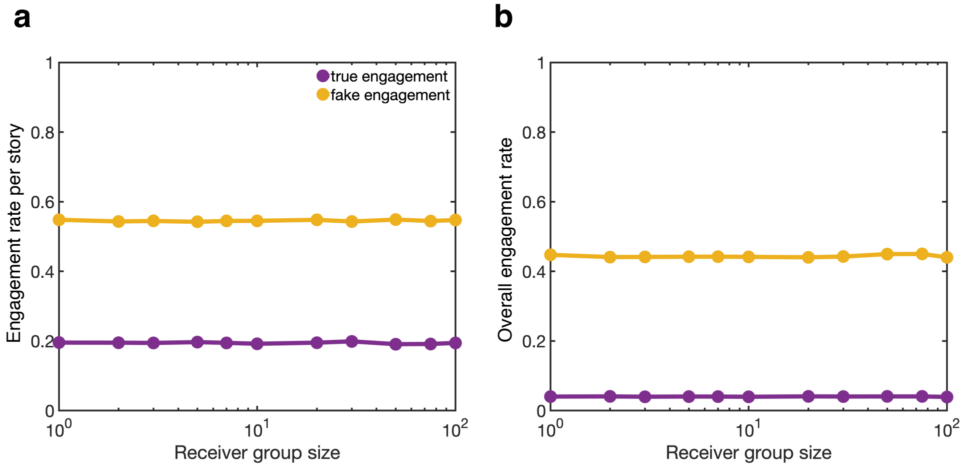

Figure S10: **Homogenous receiver groups** – a) A group of receivers optimizing in response to a single transmitter, under the same process as described for Figure S3, with the extension that that optimization occurs at the level of the group rather than the individual. We vary the size of the receiver group (x-axis) and calculate the equilibrium level of engagement with fake (orange) and true (purple) stories. b) We also calculate the equilibrium level of overall engagement. In all cases receiver mutations were local (see SI Section 1) and we set receiver payoffs $\pi_t = 2$ and $\pi_f = -1$. Here misinformation sites use a fixed linear responsive strategy $\mathbf{r} = \{1.0, 0.1, -0.9, -0.1\}$ see SI Section 1

Next we explored the effect of microtargetting on receiver engagement with misinformation in the face of a responsive transmitter. The engagement dynamics between a single transmitter and single receiver corresponds to the case of direct micro-targeting by a transmitter, in the sense that the transmitter makes their decision about what type of news to share (e.g. by promoting stories

on social media) with a receiver based solely on the engagement (or not) of that specific receiver. In reality, this degree of precision is unrealistic, but as we have shown above (Figure S9) the same effects also hold when a transmitter's audience is a group of receivers who employ similar behavioral strategies.

In order to explore how micro-targeting impacts the engagement per story and overall engagement habits of receivers interacting with a responsive misinformation strategy, we define the strength of micro-targeting as $M = 1/G$, where G is the number of independent individuals or groups to whom the transmitter promotes the same news. Here a "group" refers to a newsfeed, a social media page, or distribution list to which the transmitter promotes content. A group may contain only a single individual, or be composed of many people who share the same interest. When a transmitter promotes the same content to many such groups ($M \ll 1$), each group updates their engagement independently in response, and so the strength of micro-targeting is low: a change in the behavior of any one group has a small impact on the overall engagement with the transmitter (Main Text Eq. 2), so the transmitter is not very responsive to a given receiver. When the strength of micro-targeting is high (e.g. when there is only one transmitter and one receiver, $M = 1$) the transmitter is highly responsive to the engagement pattern of a given receiver.

Figure S11 shows the effect of varying the degree of micro-targeting available to the transmitter. We find that when micro-targeting is low the difference in engagement between false versus true stories declines, until the apparent attractiveness of misinformation disappears entirely. Thus the ability to target news stories at specific groups of receivers, either by news sites directly or by social media algorithms, can substantially contribute to the apparent attractiveness of false news stories.

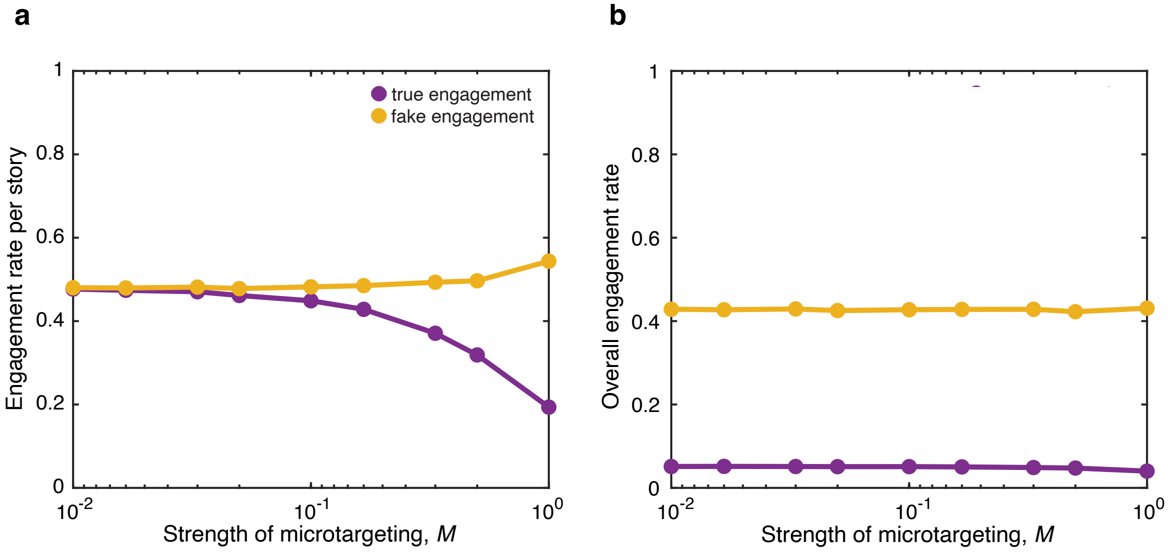

Figure S11: **Micro-targeting facilitates engagement with misinformation** a) A single misinformation site micro-targeting multiple independent receivers. The strength of micro-targeting M (x-axis) quantifies the responsiveness of the transmitter to each of the target groups of receivers (see main text). Each target receiver group is assumed to independently optimize their engagement strategy as described previously (Figure S3). The misinformation site uses the same transmission strategy for all the groups included in their targeting. The less precise the targeting of the transmitter (lower values of M), the smaller the difference in engagement between true and false news stories. b) However, the overall engagement probability remains unchanged with M. In all cases receiver strategic mutations are local (see SI Section 1) and receiver payoffs are $\pi_t = 2$ and $\pi_f = -1$. Other parameter choices are explored in the SI. Here the misinformation site uses a fixed linear responsive strategy $\mathbf{r} = \{1.0, 0.1, -0.9, -0.1\}$ see SI Section 1

3.7 The effect of different types of receiver attention

We studied the effects of varying different types of receiver attention to news content and to transmitter behavior. In the analysis presented in Figure S3 of the main text we assume that receivers are inattentive to both prior experience with a transmitter and prior information about the veracity of each headline, i.e. $a_0 = a_1 = 0$ in Eq. 1. In Figure S12 we repeat the same analysis assuming receivers are attentive to past experience, i.e. $a_1 = 1$ in Eq. 1. We see that, as in Figure S3, the most successful misinformation strategies tend to produce greater overall engagement and engagement per story with fake stories than with true. Conversely, the most successful mainstream strategies produce more engagement per story and overall engagement with true stories than false.

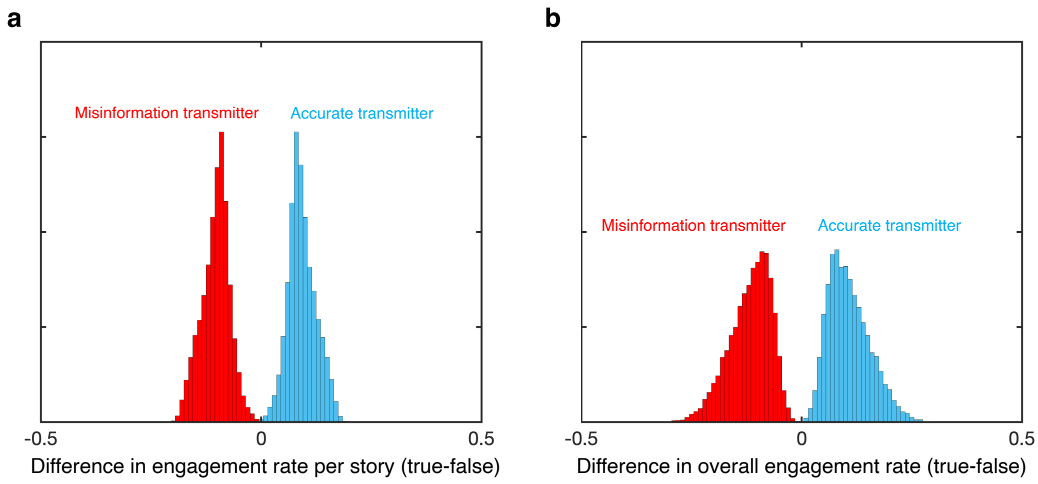

c

	Accurate transmitters	Misinformation transmitters
Engagement rate per story (true)	0.58 (0.57-0.59)	0.48 (0.42-0.53)
Engagement rate per story (false)	0.48 (0.42-0.53)	0.58 (0.57-0.59)
Overall engagement rate (all)	0.53 (0.51-0.56)	0.53 (0.51-0.56)
Overall engagement (true)	0.32 (0.29-0.37)	0.21 (0.17-0.25)
Overall engagement (false)	0.21 (0.17-0.25)	0.32 (0.29-0.37)

Figure S12: **Successful news transmission strategies with receiver memory** – To identify strategies that are successful at promoting engagement with true news stories (mainstream sites) or with misinformation stories (misinformation sites), we randomly drew 10^8 transmitter strategies (Eq. 2) and allowed a single receiver, incentivized to engage with true news, to optimize their engagement strategy (see SI Section 1) over the course of 10^4 interactions. The receiver was assumed to be inattentive to accuracy ($a_0 = 0$) but attentive to past stories ($a_1 = 1$) and optimization was assumed to be noisy ($\sigma = 1$). a) We identified the transmitter strategies that successfully promote engagement with true news stories (mainstream sites, blue), i.e those that produce a true news engagement probability within the 90th percentile from the 10^8 transmitter strategies considered, as well as $v_f < 0.5$. Similarly, we identified the transmitter strategies that successfully promote engagement with misinformation (misinformation sites, red), i.e those that produce a misinformation engagement probability within the 90th percentile, as well as $v_f > 0.5$. We plot the difference between engagement with true and fake stories, $v_{tc}/v_t - v_{fc}/v_f$, for all mainstream and misinformation sites. Mainstream site strategies induce engagement with accurate stories while misinformation site strategies induce the opposite effect. b) We also report the difference in overall engagement with true stories, v_{tc}, and overall engagement with fake stories v_{fc}. c) Summary of the engagement per story rates per story and overall engagement rates for mainstream and misinformation site transmission strategies. In all cases receiver mutations were local and we set receiver payoffs $\pi_t = 2$ and $\pi_f = -1$.

We also calculate the regions FI, FII and FIII for the effect or transmitter preference on receiver engagement patterns in the case that receivers use memory-1, i.e. $a_1 = 1$ (Figure S13). We see qualitatively similar results to the regions in Figure 3. Surprisingly, we find that the region FII, in which receiver engagement reflects transmitter preference, despite receivers preferring accurate stories, is slightly larger than the case $a_1 = 0$.

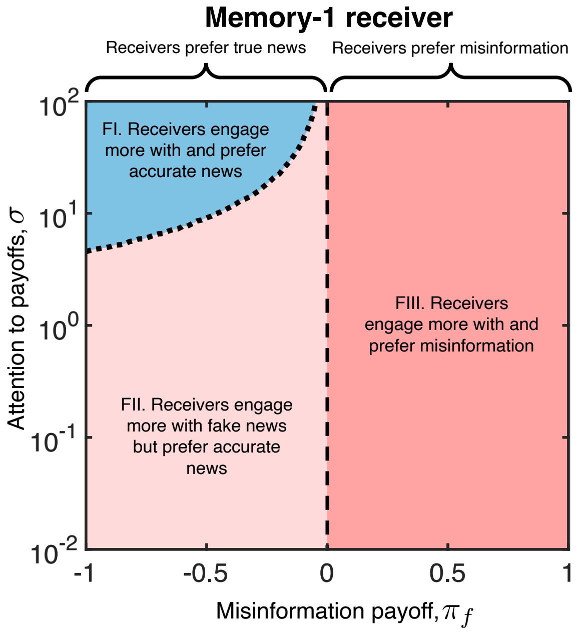

Figure S13: **Effect of memory, receiver preferences and attentiveness on engagement** – We calculated the engagement patterns among receivers optimizing under different preferences (where we have set $\pi_t = -\pi_f$), and different levels of attention, σ in the same way as for main text Figure 3, for a receiver with memory, $a_1 = 1$. We see a qualitatively similar pattern to the case with no memory, $a_1 = 0$, with an increased region FII. In all cases receiver strategic exploration was local, with $a_0 = 0$, with transmitter attention set at $\sigma = 100$. Optimization occurred over 10^4 time-steps using ensembles of 10^3 replicates for each value of $\{\pi_f, \sigma\}$ (see SI section 1).

The distribution of engagement per story and overall engagement for all 10^8 transmitter strategies when receivers employ memory of their previous interaction ($a_1 = 1$) is shown in Figure S14. We see that the distribution of engagement is narrower than in Figure S3, when receivers are inattentive to memory, while the distribution of overall engagement follows a similar distribution in both cases.

We also considered (Figure S15) the effects of receivers conditioning their behavior on memory of previous interactions beyond simply the immediately preceding encounter. We assumed that

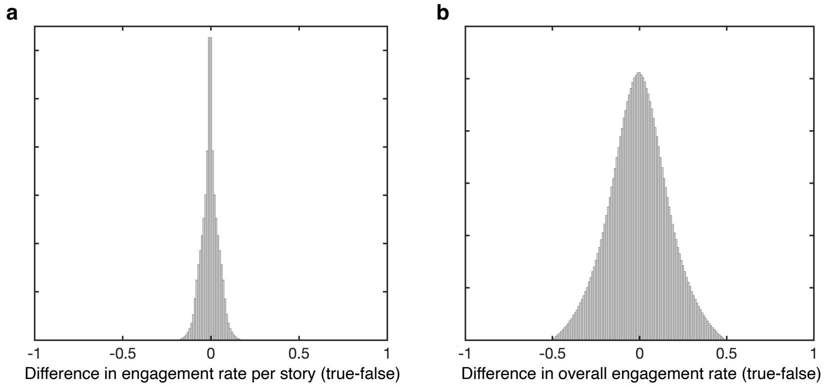

Figure S14: **Full distribution of engagement per story and overall engagement with receiver memory** – Shown are the distributions of the difference in engagement per story (a) and overall engagement (b) with true vs fake stories for 10^8 transmitter strategies. We define successful misinformation strategies as those with $v_f > 0.5$ and misinformation engagement, v_{fc}/v_f in the 90th percentile among all observed strategies. Similarly we define successful mainstream strategies as those with $v_t > 0.5$ and misinformation engagement, v_{tc}/v_t in the 90th percentile among all observed strategies.

receivers treated a transmitter as having shared misinformation with them when employing their strategy $\mathbf{p}$ (Eq. 1) if the transmitter has shared a fake story in *any* of the last m rounds. We show engagement per story and overall engagement as a function of such memory in Eq. S7, where we see that longer memory of this type has only a weak effect on engagement per story and overall engagement, and does not by itself reverse the trend of successful misinformation sites generating higher engagement per story and overall engagement with fake vs true stories among inattentive consumers.

We also studied the effect of varying attention to headline accuracy in Eq. 1, against successful misinformation promoting transmitter strategies. We varied attention to accuracy, a_0, memory of past interactions, a_1 and attention to payoffs, σ among receivers. We also considered the case where receivers both prefer true news (as in the main text) and the case where they prefer misinformation, i.e. derive benefit π_t from interacting with misinformation and pay cost π_f for interacting with true news (Figure S16).

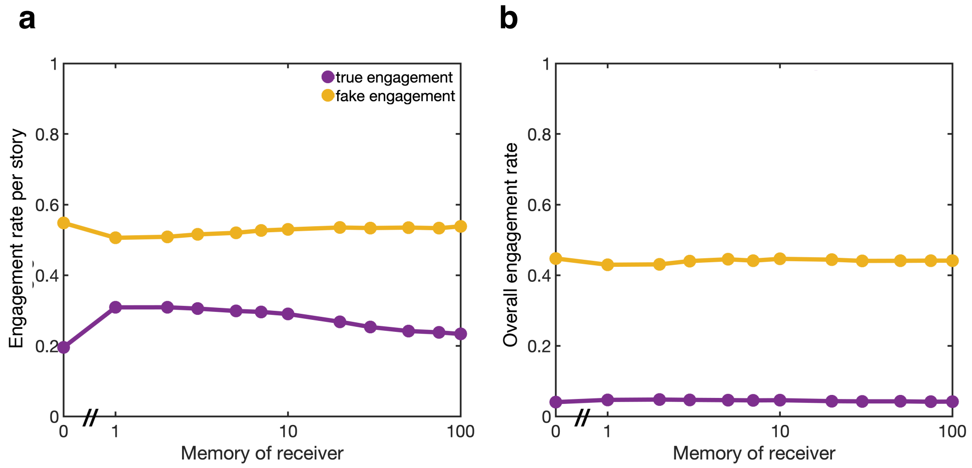

Figure S15: **Receiver memory length** – a) A single receiver optimizing in response to a single transmitter, under the same process as described for Figure S3. The receiver uses memory of preceding rounds to decide whether the transmitter shares true stories (and so is labelled t) or fake stories (and so is labelled f). We assume that if the receiver identifies any item of misinformation in their memory of the transmitter's shared stories, they label the transmitter as fake (f). We vary the length of this memory (x-axis) and calculate the equilibrium level of engagement with fake (orange) and true (purple) stories. b) We also calculate the equilibrium level of overall engagement. In all cases receiver mutations were local (see SI Section 1) and we set receiver payoffs $\pi_t = 2$ and $\pi_f = -1$. Here misinformation sites use a fixed linear responsive strategy $\mathbf{r} = \{1.0, 0.1, -0.9, -0.1\}$ see SI Section 1

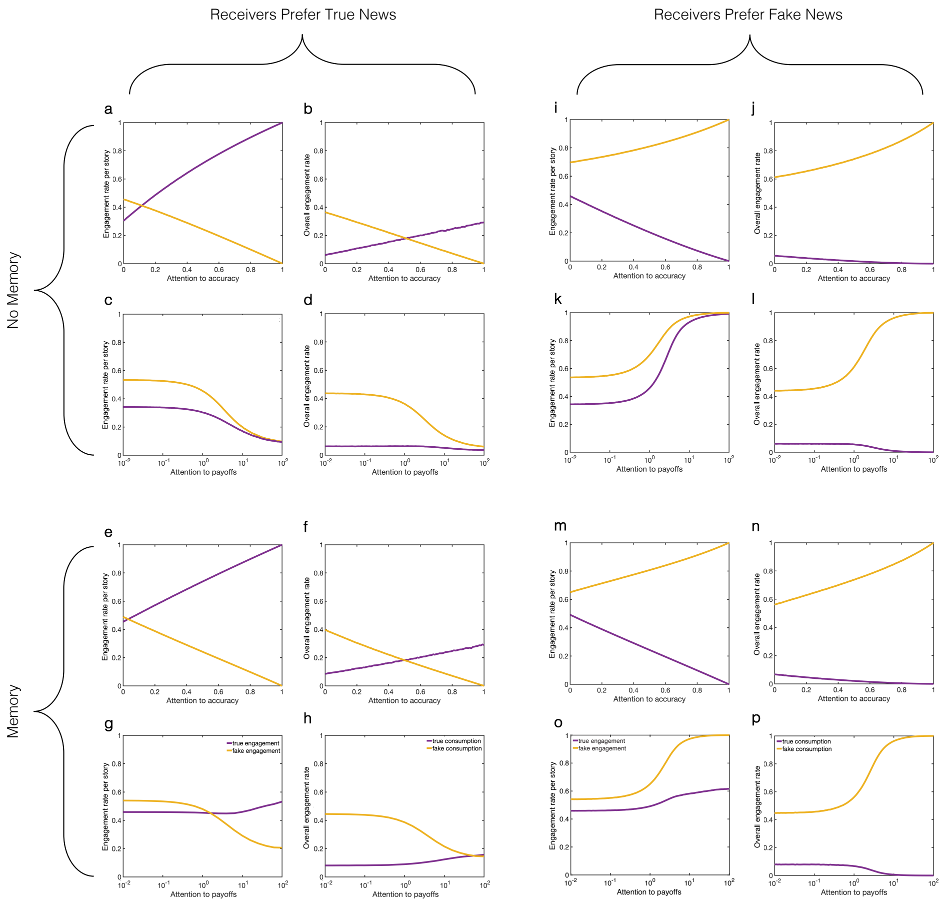

Figure S16: **Attention and news engagement** – We randomly drew 10^5 misinformation strategies (see SI Section 1) and allowed receivers to optimize as described in Figure S3. (a-b) When receivers have no memory $a_1 = 0$, and pay little attention to long-term payoffs, $\sigma = 1$, but prefer accurate information, we increased attention to accuracy in the optimization process (a_0) (x-axis), and calculated a) the average probability of engagement with true (purple) and fake (orange) stories. As attention to accuracy increases, overall engagement per story with fake stories decreases to zero, while engagement per story with true stories increases. b) Similarly, overall engagement probability with fake stories decreases with increasing attention to accuracy, while overall engagement with true stories increases. (c-d) When receivers have no memory $a_1 = 0$, and no attention to accuracy, $a_0 = 0$, but prefer accurate information, we increased attention to long-term payoffs in the optimization process (σ) (x-axis), and calculated c) the average probability of engagement with true (purple) and fake (orange) stories. As attention to payoffs increases, overall engagement with true and fake stories decreases to zero. d) Similarly, overall engagement probability with true and fake stories decreases with increasing attention to payoffs. (e-h) Shows the same plots as in (a-d) except with memory of past interactions, i.e. $a_1 = 1$. (e-f) show similar patterns as (a-b) while (g-h) shows increasing engagement per story and overall engagement for true news (purple) with increasing attention to payoffs (σ). (i-l) Shows the same plots as in (a-d) except with receivers preferring misinformation, i.e. gaining benefit π_t from interacting with a fake story and paying a cost π_f from interacting with a true story. (i-j) show opposite patterns to (a-b) while (k-l) show k) increasing engagement for both true and misinformation engagement with attention to payoffs, but l) declining overall engagement with true news. (m-p) Shows the same plots as in (e-h) except with receivers preferring misinformation, i.e. gaining benefit π_t from interacting with a fake story and paying a cost π_f from interacting with a true story. (m-n) show opposite patterns to (e-f) while (o-p) show opposite patterns to (g-h). In all cases we set attention to payoffs $\sigma = 1$ and receiver payoffs $\pi_t = 2$ and $\pi_f = -1$ unless otherwise stated.

We see that in many cases increasing attention not only reduces engagement per story and overall engagement among receivers who prefer true news (Figure S16 panels a-h) it can also reverse the apparent preference for misinformation induced by responsive transmitter strategies. However increased attention tends to have the opposite effect on engagement per story and overall engagement when receivers prefer misinformation (Figure S16 panels i-p).

Finally we calculated the regions FI, FII and FIII for different rates of attention a_0 to headline veracity (Figure S7). As expected, increasing a_0 increases the size of region FI and decreases the size of region FII, i.e. receivers become increasingly likely to engage with news that reflects their own preference rather than that of the transmitter.

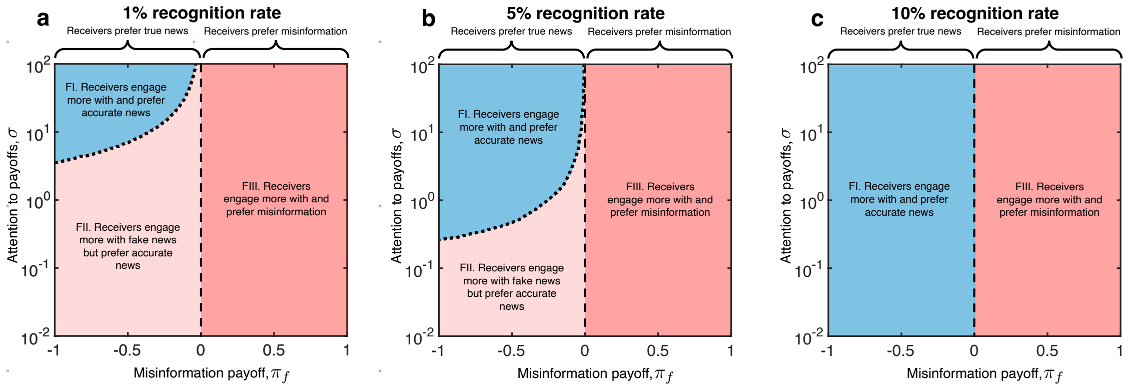

Figure S17: **Effect of prior knowledge, receiver preferences and attentiveness on engagement** – We calculated the engagement patterns among receivers optimizing under different preferences (where we have set $\pi_t = -\pi_f$), and different levels of attention, σ in the same way as for main text Figure 3, for different levels of attention to headline veracity, a_0. a) When $a_0 = 0.01$, meaning 1% of false stories can be identified from their headline, we see little change in the size of region FII as compared to Figure S13 (for which $a_0 = 0$). b) When $a_0 = 0.05$, meaning 5% of false stories can be identified from their headline, we see a significant increase in the size of region FII as compared to Figure S13. c) When $a_0 = 0.1$, meaning 10% of false stories can be identified from their headline, region FII vanishes entirely, and receiver engagement patterns always match their preferences. In all cases receiver strategic exploration was local, with memory $a_1 = 1$, with transmitter attention set at $\sigma = 100$. Optimization occurred over 10^4 time-steps using ensembles of 10^3 replicates for each value of $\{\pi_f, \sigma\}$ (see SI section 1).

3.8 The effect of multiple news transmitters on consumer behavior

We studied the effect of multiple transmitters, with a receiver facing a single misinformation site within in a population of otherwise accurate news transmitters (Figure S18). We assume that the receiver optimises in response to a pool of stories produced by *all* of the transmitters simultaneously. Note that this is a simple model of receiver "choice", in which the receiver makes no attempt to distinguish between sources, but simply reacts to the total pool of stories. A scenario in which receivers treat different sources differently is captured by the dynamics of a single transmitter and receiver. However, we do not attempt to model a scenario in which receiver's engagement with one transmitter depends on the output of another (as would happen, for example, if the receiver was deciding which daily newspaper to buy).

We see that under our model of multiple transmitters the receiver tends to engage with more misinformation from the misinformation site as the population size of mainstream sites grows. This makes intuitive sense, because the relative contribution of the misinformation site to the receiver's payoff necessarily declines as the population grows, and so avoiding misinformation becomes less important to receiver payoff.

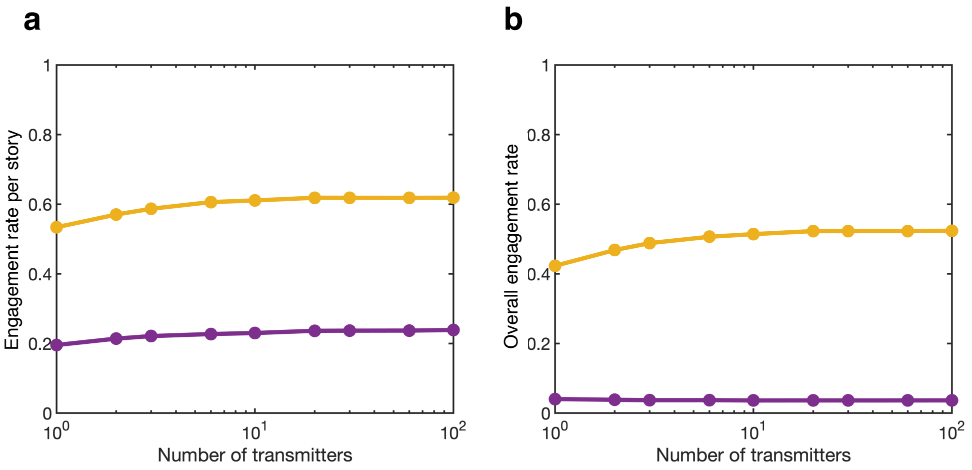

Figure S18 **Groups of transmitters** – a) A single receiver optimizing in the face of a population of transmitters, under the same process as described for Figure S3. The transmitter population consists of a single misinformation site and $N-1$ mainstream sites. Increasing the number of mainstream sites increases engagement with true (purple) and fake (orange) news stories from the misinformation transmitter as well as b) overall misinformation engagement. In all cases receiver mutations were local (see SI Section 1) and we set receiver payoffs $\pi_t = 2$ and $\pi_f = -1$. Other parameter choices are explored in the SI. Here misinformation sites use a fixed linear responsive strategy $\mathbf{r} = \{1.0, 0.1, -0.9, -0.1\}$ and mainstream sites use a fixed linear responsive strategy $\mathbf{r} = \{0.9, 0.0, 0.1, 0.9\}$ see SI Section 1

3.9 The effect of supply and demand

We studied the effects consumer demand on transmitter supply of misinformation. To do this we varied receiver payoff on overall engagement and engagement per story, shown in Figure S19 and Figure S20. In Figure S19 we fix the cost π_f of consuming misinformation, and vary the benefit π_t of consuming true news, for different levels of attention to payoff, σ. We see that increasing benefits of true news often has only weak effects on overall engagement and engagement per story, but do tend to increase overall levels of both once benefits become sufficiently large.

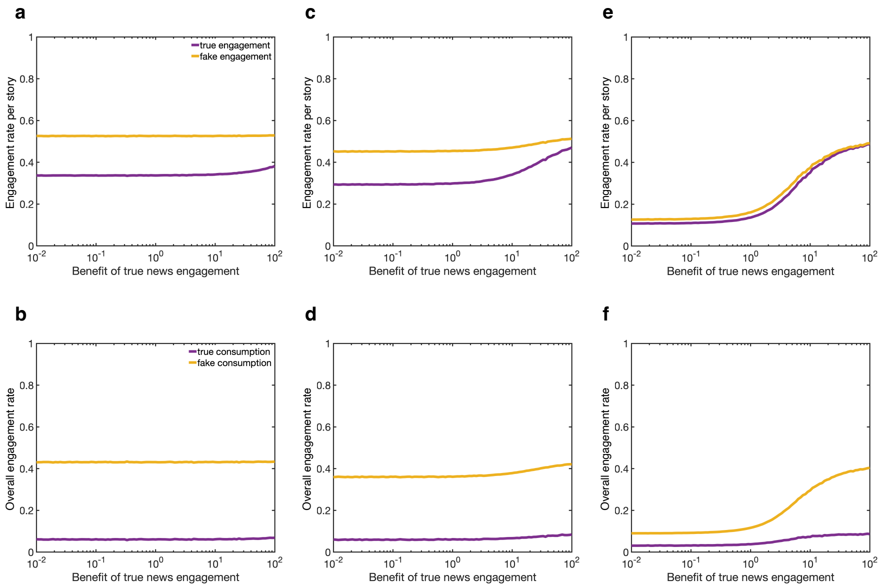

Figure S19: **Varying benefit of true news engagement** – We randomly drew 10^5 responsive misinformation strategies (see SI Section 1) and allowed receivers to optimize as described in Figure S3. a) When attention to payoff is low, ($\sigma = 0.1$), cost of engaging with misinformation is fixed, ($\pi_f = -1$), and receivers have no memory and no attention to story accuracy, $a_0 = a_1 = 0$, we increased the benefit of engaging with true news stories π_t in the optimization process (x-axis), and calculated the average probability of engagement with true (purple) and fake (orange) stories. As attention to payoffs increases, engagement per story slowly increases and the difference in engagement with true and fake stories declines. b) Similarly, overall engagement when attention to payoffs is low ($\sigma = 0.1$) remains close to constant. c) When attention to payoffs is moderate ($\sigma = 1$) the same pattern holds for engagement per story while d) overall engagement slowly increases, and the difference between overall engagement with true and misinformation also increases. e) When attention to payoffs is strong ($\sigma = 10$) there is little difference between true and misinformation engagement, and engagement per story increases with π_t while f) overall engagement increases, and the difference between overall engagement with true and misinformation also increases. Unless otherwise stated parameters are the same as in Figure S3.

In Figure S20 we fix the benefit π_t of consuming true news, and vary the cost π_f of consuming misinformation, for different levels of attention to payoff, σ. We see that increasing costs of

misinformation has a strong effect on overall engagement and engagement per story, reducing both close to zero once costs become sufficiently large. This result is consistent with our game-theoretic expectation that higher costs require a misinformation sharing strategy to include ever higher levels of true news to make engagement worthwhile for the receiver.

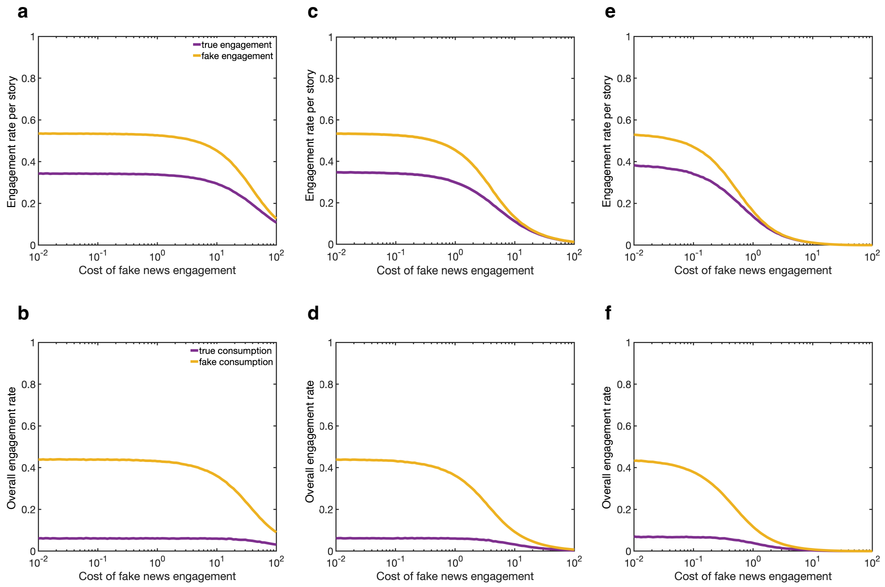

Figure S20: **Varying cost of true news engagement** – We randomly drew 10^5 responsive misinformation strategies (see SI Section 1) and allowed receivers to optimize as described in Figure S3. a) When attention to payoff is low, ($\sigma = 0.1$), benefit of engaging with true news is fixed, ($\pi_t = 1$), and receivers have no memory and no attention to story accuracy, $a_0 = a_1 = 0$, we increased the cost of engaging with misinformation stories π_f in the optimization process (x-axis), and calculated the average probability of engagement with true (purple) and fake (orange) stories. As attention to payoffs increases, engagement per story slowly decreases and the difference in engagement with true and fake stories declines. b) Similarly, overall engagement when attention to payoffs is low ($\sigma = 0.1$) declines as π_f increases. c) When attention to payoffs is moderate ($\sigma = 1$) the same pattern holds for engagement per story and d) overall engagement. e) When attention to payoffs is strong ($\sigma = 10$) both engagement per story and f) overall engagement decline to zero once $\pi_f < -1$. Unless otherwise stated parameters are the same as in Figure S3.

4 Supplementary information on experiments

Here we provided additional details and analysis of our experimental findings.

4.1 Headline selection and engagement data

We studied 20 mainstream and 20 misinformation sources, used in previous studies of misinformation online [10]. We selected the most recent 20 unique news headlines with engagement data available for Facebook via CrowdTangle. We excluded stories whose headlines did not constitute news (e.g. a quiz). We also studied the relationship between source trust and patterns of engagement without a priori groupings of mainstream and misinformation sites (Figure S21). To do this we used trust ratings from [5].

We gathered engagement data via CrowdTangle on the most recent 20 news headlines for each news source as available on November 22nd, 2020. This information was gathered by generating a historical report for each news site and gathering the 20 most recent news headlines in the database. The engagement information recorded for each story is the number of Facebook interactions reported by CrowdTangle at the time. This number accounts for likes, comments and shares of posts that contain the URL for the article across the CrowdTangle database.

The list of headlines for each source, the headline accuracy rating, the publication date of the story and the Facebook engagement information recovered via CrowdTangle is available via github here.

4.2 Meta-analysis

We performed a meta-analysis on the results of linear regressions of engagement against accuracy, for the 20 misinformation and 20 mainstream sites. In order to determine whether there was a negative correlation between engagement and accuracy for misinformation sites, we performed Fisher's combined test, using the p-values resulting from a single-tailed t-test from a regression of engagement against accuracy. we used the same procedure to determine whether there was a positive correlation between engagement and accuracy for mainstream news sites.

To determine whether there was significant heterogeneity among misinformation or mainstream

sites we calculated the between study variance τ^2_{DL} using the DerSimonian and Laird [4] moment-based estimate, and calculated the proportion of total variance attributable to between study effects, I^2, as well as the degree of homogeneity Q_{RE} under a random-effects model [7]. These results are summarized in Table S3 where we test regressions of $\log_{10}$-engagement vs accuracy, using both unstandardadized and standardized data, as well as regression using the rank of engagement and accuracy for each site. In all cases we find significant negative correlation for misinformation and significant positive correlation for mainstream sites with $p < 0.01$. However we find no significant between site heterogeneity for either I^2 or Q_{RE} with $p < 0.05$.

	Mainstream sites			Fake news sites		
	$\hat{\mu}_{RE}$	Q_{RE}	I^2	$\hat{\mu}_{RE}$	Q_{RE}	I^2
Unstandardized	0.08* (0.02,0.13)	18.3	20%	-0.07* (-0.14, 0.01)	18.8	35.5%
Standardized	0.13* (0.03, 0.23)	18.5	24.1%	-0.11* (-0.22,-0.01)	18.4	29.4%
Ranked	0.12* (0.03, 0.22)	18.7	13.5%	-0.1* (-0.2, -0.01)	18.8	23.3%

Table S3: **Meta analysis** – The mean effect size $\hat{\mu}_{RE}$ for correlations with weights from a random effect model, as well as measures between study heterogeneity I^2 and Q_{RE}. Significance is indicated with an asterisk (*).

4.3 Experiment A: Empirical patterns of misinformation engagement (MTurk).

In addition to the results for Experiment A presented in main text Figure 3, we also assessed each story's perceived accuracy by recruiting 1,000 American participants from Amazon Mechanical Turk to rate the accuracy of 20 headlines (yielding a total of 20,000 accuracy ratings), which has been shown to produce good agreement with the ratings of professional fact-checkers via the wisdom of crowds [3]. We see that mainstream news sites, which we assume seek to promote accurate information, tend to share headlines with higher accuracy ratings than fake news sites ($p < 0.001$, Figure S21a). Importantly, however, both mainstream and fake news sites show wide variation in perceived headline accuracy. Thus, there is substantial overlap between the content produced by the two kinds of sites, with many articles from fake news sites being rated as more accurate than many articles from mainstream sites.

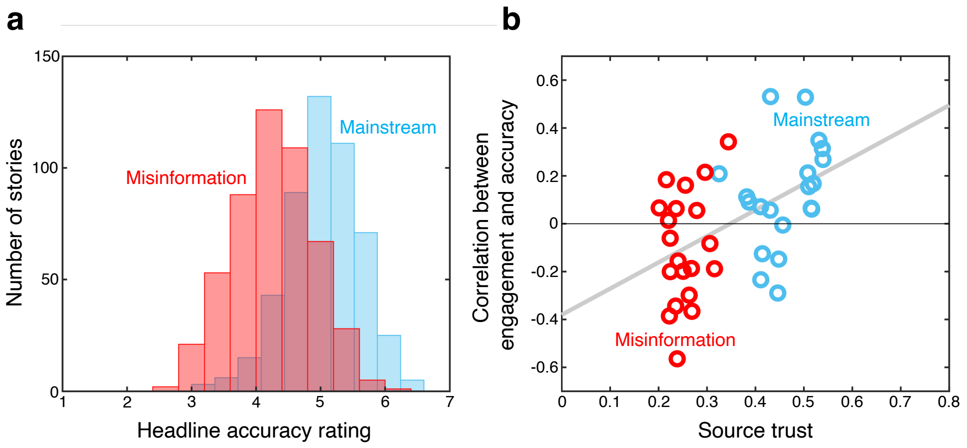

Figure S21: **Empirical patterns of news engagement and accuracy, for mainstream and misinformation sites** – We selected 20 mainstream and 20 fake news sites identified in previous studies of misinformation [10]. Using Crowdtangle we selected the most recent 25 news stories for which engagement data on Facebook was available (see Methods). We then recruited American subjects from Amazon Mechanical Turk to assess headline accuracy (20 ratings per headline). a) Distribution of accuracy ratings for mainstream sites (blue, mean=5.05, SD=0.56) and mainstream sites (red, mean=4.27, SD=0.62). As expected stories from misinformation sites are rated as significantly less accurate than stories from mainstream sites ($p < 0.001$, two-sample t-test). b) Regression of individual site trust ratings [5] against standardized accuracy-engagement regression coefficient (see Methods). More trusted sites tend to produce higher engagement with accurate information, while less trusted sites tend to produce higher engagement with less accurate information ($p = 0.0008$, $r^2 = 0.25$).

Table S4 shows the correlation between accuracy and engagement (as shown in main text Figure 4).

Site	Correlation coefficient	Type
yournewswire	-0.5643	Misinformation
angrypatriotmovement	-0.3855	Misinformation
socialeverythings	-0.3699	Misinformation
clashdaily	-0.3424	Misinformation
freedomdaily	-0.2984	Misinformation
nypost	-0.2832	Mainstream
bostonglobe	-0.2335	Mainstream
newsbreakshere	-0.2011	Misinformation
beforeitsnews	-0.1994	Misinformation
downtrend	-0.1985	Misinformation
usherald	-0.1881	Misinformation
dailybuzzlive	-0.1851	Misinformation
realnewsrightnow	-0.1539	Misinformation
huffingtonpost	-0.1441	Mainstream
foxnews	-0.1254	Mainstream
americannews	-0.0828	Misinformation
chicagotribune	-0.0075	Mainstream
bb4sp	0.0142	Misinformation
onepoliticalplaza	0.0379	Misinformation
conservativedaily	0.0545	Misinformation
latimes	0.0574	Mainstream
nytimes	0.061	Mainstream
msnbc	0.0648	Mainstream
react365	0.0655	Misinformation
notallowedto	0.0713	Misinformation
nydailynews	0.0713	Mainstream
dailymail	0.0896	Mainstream
aol	0.1121	Mainstream
usatoday	0.1575	Mainstream
cnn	0.1675	Mainstream
whatdoesitmean	0.1821	Misinformation
sfchronicle	0.2093	Mainstream
yahoo	0.2097	Mainstream
thenewyorkevening	0.2172	Misinformation
washingtonpost	0.2686	Mainstream
abcnews	0.3153	Mainstream
channel24news	0.3375	Misinformation
cbsnews	0.35	Mainstream
bbc	0.5288	Mainstream
wsj	0.5313	Mainstream

Table S4: **Correlation between accuracy and engagement**

4.4 Experiment B: Empirical patterns of receiver preference, with accuracy elicited (Lucid).

Experiment B is detailed in the Methods section of the main text. We asked participants to assess the likelihood of sharing and the likelihood of clicking of 40 headlines. Unlike Experiment B, however, we did *not* ask questions related to the accuracy of the displayed contents. Once participants completed the task, we asked them which domains they regularly used for news. The study was approved by MIT COUHES with the same protocol (1806400195).

The observed preferences for the general population sample (Figure S22) are consistent with those observed among Twitter users (Experiment C, see Figure 4). There is a strong positive correlation between perceived accuracy and willingness of readers to click or share an article ($p < 0.001$). This holds for those recruited from the general population regardless of their self-reported use of misinformation sites. The results are qualitatively equivalent when using objective accuracy (as measured by professional fact-checkers; $p < 0.001$ for both outcomes across both groups).

4.5 Supplemental Experiment: Empirical patterns of receiver preference, without accuracy elicited (Lucid).

Similar to Experiment B detailed in the main text Methods, here we asked participants to assess the likelihood of sharing and the likelihood of clicking of 40 headlines. Unlike Experiment B, however, we did *not* ask questions related to the accuracy of the displayed contents. Once participants completed the task, we asked them which domains they regularly used for news. The study was approved by MIT COUHES with the same protocol (1806400195).

Participants. From 27 April 2022 to 3 May 2022, we recruited 891 participants from Lucid but excluded: 270 who failed two trivial attention checks at the study outset, 75 who reported not having at least one social media account, and 30 who did not provide data on the use of at least one of the 60 listed domains for news. Thus, our final sample consists of 516 observations with valid records for the main analysis. The sample included 233 males and 258 females, with a mean age of 48.87 years (min. 18; max. 90). Median completion time was 10 minutes and 17 seconds.

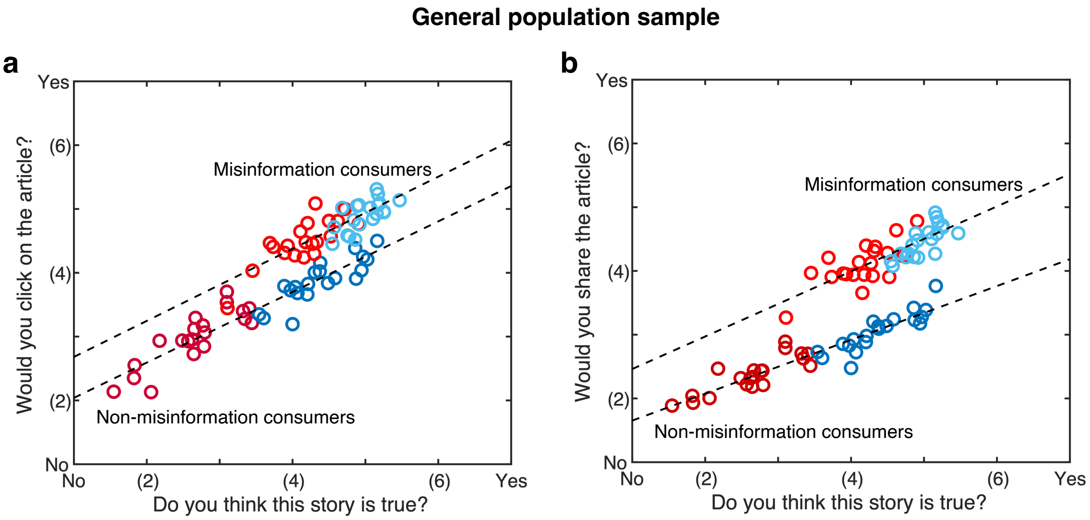

Figure S22: **Misinformation consumers prefer to engage with accuracy news.** We selected 20 mainstream (blue) and 20 misinformation (red) stories identified in previous studies of misinformation [10]. We then recruited American subjects from Lucid, to assess the accuracy of 10 headlines of each type. Each participant was then asked to rate their willingness to click on and share the article associated with the headline. a) A general population sample recruited from Lucid contained 124 participants who indicated engagement with misinformation sites (light colors) and 387 who indicated only engagement with mainstream sites (dark colors). Both fake and non-misinformation consumers show a strong positive correlation between perceived accuracy and willingness to click (misinformation consumers: $\beta = .662, z = 18.13, p < 0.001$; non-misinformation consumers: $\beta = .590, z = 29.45, p < 0.001$; difference between misinformation and non-misinformation consumers, $\beta = .072, z = 1.83, p = 0.068$), and between perceived accuracy and willingness to share (misinformation consumers: $\beta = .682, z = 19.86, p < 0.001$; non-misinformation consumers: $\beta = .585, z = 22.75, p < 0.001$; difference between misinformation and non-misinformation consumers, $\beta = -.097, z = -2.39, p = 0.017$).

Materials. We used the same set of 40 news "cards" and 60 domains as Experiment B. We then asked participants to : i) evaluate 20 cards, drawn randomly from the set of 40, on the likelihood of sharing (i.e. "If you were to see the above headline online, would you share it?", seven-point scale from "Definitely NO" to "Definitely YES"), and likelihood of clicking (i.e. "If you were to see the above headline online, would you click on it to read the article?", seven-point scale from "Definitely NO" to "Definitely YES") of the information presented; ii) select which of the 60 domains they regularly use for news (with this information, we classified participants as misinformation media users if they selected at least one domain pre-identified as a misinformation outlet). Similar to Experiment B, the study concluded with a list of 20 exploratory items.

Results Our pre-registered main analysis was to restrict to data from participants who indicate regularly using one or more misinformation sites for news, and run 2 linear regressions with two-way clustered standard errors clustered on subject and headline, predicting sharing intentions or click intentions as the dependent variable and taking the average accuracy rating of the given headline collected in Experiment B as the independent variable. Consistent with the results of Experiment B, in the Supplemental Experiment we find that fake new site users show a significant positive relationship between out-of-sample perceived accuracy and intent to both share ($\beta = .048, t = 2.96, p = 0.003$) and click ($\beta = .047, t = 2.26, p = 0.024$). It is unsurprising that the strength of these correlations is substantially weaker than in Experiments B and C, because here we use population-average perceived accuracy from another study, rather than subject-specific perceived accuracy (and thus our predictor is measured with much more noise). Consistent with this interpretation, the associates are much higher stronger when averaging clicking and sharing intentions for each headline across subjects (thus matching the way perceived accuracy is calculated) and conducting a post hoc analysis at the headline level (see Figure S23). Be that as it may, the fact that we continue to observe significant positive associations in the Supplemental Experiment indicates that our key result from the main experiments is not simply an artifact of having asked participants to evaluate accuracy prior to making their sharing and clicking responses.

We also pre-registered a secondary analysis comparing participants who engage with misinformation sites to participants who do not, by z-scoring each variable within user type and re-running the 2 main models including all subjects and adding a dummy for not visiting any misinformation sites, as well as the interaction between accuracy rating and this dummy. For sharing intentions, the association with out-of-sample perceived accuracy is marginally higher for non-fake-news-site users ($\beta = .028, z = 1.82, p = 0.069$); and significantly higher for clicking intentions ($\beta = .028, z = 1.82, p = 0.022$). The associations for both outcomes and both types of users are shown at the headline-level in Figure S23.

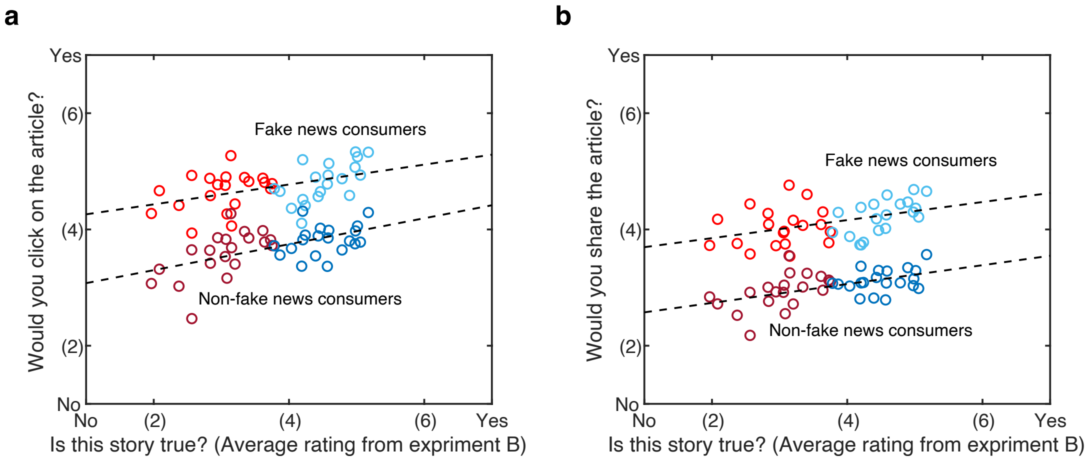

Figure S23: **The preferences of misinformation consumers.** We repeated Experiment B as described above, without prompting for accuracy. a) A general population sample recruited from Lucid contained 125 participants who indicated engagement with misinformation sites (light colors) and 421 who indicated only engagement with mainstream sites (dark colors). Both fake and non-misinformation consumers show a strong positive correlation between accuracy and willingness to click, (non-fake consumers: $r^2 = 0.31$, fake consumers: $r^2 = 0.20$), and b) accuracy and willingness to share (non-fake consumers: $r^2 = 0.29$, fake consumers: $r^2 = 0.17$), where accuracy ratings used are averaged from Experiment B.

4.6 Breakdown of the demographic and political leanings of our participants

Here we present a table summarizing the age, sex, education, and political leaning of the participants in our four samples. Studies B and B bis match the expected quotas of a nationally-representative sample.

As can be seen, the study conducted on Twitter consists of a relatively older and more educated sample, with a slightly larger leaning towards the Republican party (6-point Likert scale; 1 - Strongly Democrat 6 - Strongly Republican). On the other hand, the study conducted on MTurk consists of a relatively younger and less educated sample, with a slightly larger leaning towards the Democratic party.

Study				
	A	B	B bis	C
Age bracket (Average)	38.5	47.0	48.9	57.8
18 to 24	5.4%	10.7%	10.7%	0%
25 to 34	42.8%	20.1%	18.8%	3.3%
35 to 44	22.4%	16.6%	18.0%	12.2%
45 to 54	16.5%	16.2%	15.9%	20.0%
55 to 64	10.4%	15.8%	15.7%	32.2%
Over 64	2.5%	20.7%	20.9%	32.2%
Gender				
Male	57.6%	47.3%	46.3%	50.4%
Female	42.4%	51.7%	51.3%	44.4%
Education				
At least Bachelors Degree	21.0%	36.0%	31.0%	52.1%
Political leaning (Average)	3.2	3.4	3.4	3.8
1-Strongly Democratic	17.3%	18.7%	15.7%	17.7%
2-Democratic	26.0%	14.6%	15.3%	10.1%
3-Lean Democratic	15.7%	17.1%	21.4%	7.6%
4-Lean Republican	13.1%	21.7%	23.9%	26.9%
5-Republican	17.8%	14.2%	11.6%	19.3%
6-Strongly Republican	10.1%	13.8%	12.2%	18.5%

Table S5: **Demographic breakdown of participants.**

4.7 Robustness of empirical results to partisanship

In Experiment A our finding was that misinformation sites generate a negative correlation between accuracy and engagement while mainstream sites generate a positive correlation. To do this we compared engagement data from CrowdTangle with headline accuracy ratings from a survey experiment. Here we investigate whether the partisan preferences of those rating the accuracy of headlines impact our results. To do this we repeat the same analysis (calculating the regression coefficient of standardized engagement against accuracy for each news site) using accuracy ratings only from those participants who lean Democrat or Republican. We find no significant difference between the resulting correlation coefficients among headlines from mainstream sites ($p = 0.23$, $t = 1.22$) and no significant difference between the correlation coefficients among headlines from misinformation sites ($p = 0.15$, $t = 1.46$).

In Experiments B, C, and the Supplemental Experiment, our finding was that misinformation site users prefer to engage with posts they believe are accurate. Here, we conduct a series of post hoc analyses to investigate whether there are partisan differences these preferences. To do so, we replicate the main analyses for each experiment (predicting sharing or clicking intentions using perceived accurate, using linear regression with robust standard errors clustered on subject and headline) while adding a user partisanship variable (preference for the Democratic versus Republican party on a 6-point Likert scale) and the interaction between user partisanship and perceived accuracy. This interaction indicates how the relationship between perceived accuracy and the outcome differs based on user partisanship.

In Experiment B, we find no significant interaction between partisanship and perceived accuracy when predicting either sharing intentions ($\beta = -.050, z = -0.73, p = 0.464$) or clicking intentions ($\beta = -.075, z = -1.01, p = 0.311$).

In Experiment C, we find no significant interaction between partisanship and perceived accuracy when predicting sharing intentions ($\beta = .000, z = 0.01, p = 0.992$). We do find a significant negative interaction when predicting clicking intentions ($\beta = -.052, z = -2.11, p = 0.035$), such that the association is weaker for users who are more supportive of the Republican party. Nonetheless, even for maximally strong Republicans, we continue to find a strong positive association between

perceived accuracy and clicking intention ($\beta = .318, z = 3.50, p < 0.001$).

In the Supplemental Experimental, we find no significant interaction between partisanship and perceived accuracy when predicting either sharing intentions ($\beta = -.018, z = -1.38, p = 0.167$) or clicking intentions ($\beta = -.011, z = -0.83, p = 0.406$).

Thus we do not find evidence that the positive association between perceived accuracy and engagement is unique to one political party or the other.

4.8 Experimental interface

Experiment A. In Experiment A we used Crowdtangle to gather the 25 most recently available news stories from 40 media outlets (i.e. 1,000 articles; 500 posted by 20 misinformation sites and 500 posted by 20 mainstream sites), along with the headline, lede, date of publication, link and level of engagement. See Supplementary Information section 3 for additional details. We then used this data to present the participants outlined above with 20 article headlines and ledes, drawn randomly from within one of the two media outlet subsets, and asked them to assess the accuracy of the information they were faced with. Specifically, they were asked "Do you think this story is true?", to which they responded on a seven-point scale from "Definitely NO" to "Definitely YES". The study concluded with seven demographic questions (age, gender, education, political conservativeness on social and economic issues, political position, and political preference) and a section to leave comments. An illustrative example is shown in Figure S24.

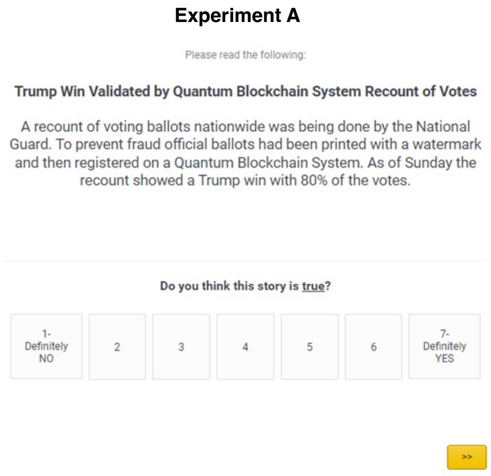

Figure S24: **Interface for Experiment A.**

Experiment B & C. We identified a pool of 40 news ”cards” (i.e. representations of Facebook posts with an image, headline, and a source; balanced on veracity and partisan lean) and a list of 60 domains regularly used for news (20 identified as mainstream, 20 as hyper-partisan, and as 20 fake by Pennycook and Rand, 2019 [10]). We then asked participants to : i) evaluate 20 cards, drawn randomly from the set of 40, on the accuracy (i.e. “Do you think this story is true?”, seven-point scale from “Definitely NO” to “Definitely YES”), likelihood of sharing (i.e. “If you were to see the above headline online, would you share it?”, seven-point scale from “Definitely NO” to “Definitely YES”), and likelihood of clicking (i.e. “If you were to see the above headline online, would you click on it to read the article?”, seven-point scale from “Definitely NO” to “Definitely YES”) of the information presented; ii) select which of the 60 domains they regularly use for news (with this information, we classified participants as misinformation media users if they selected at least one domain pre-identified as a misinformation outlet). The study concluded with a list of 20 exploratory items. An illustrative example is shown in Figure S25.

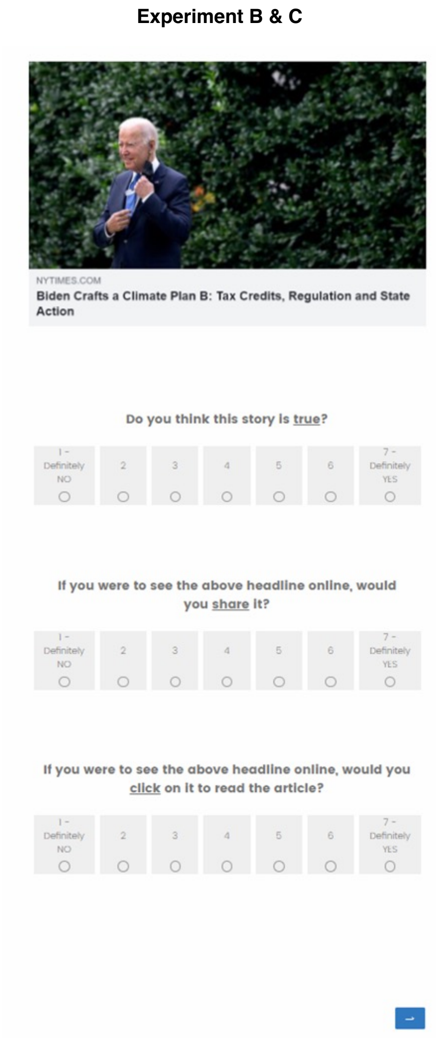

Figure S25: **Interface for Experiments B&C**

References

[1] E. Akin. What you gotta know to play good in the iterated prisoner's dilemma. *Games*, 6(3):175–190, 2015.

[2] H. Allcott and M. Gentzkow. Social media and fake news in the 2016 election. *Journal of Economic Perspectives*, 31(2):211–36, May 2017.

[3] J. Allen, A. A. Arechar, G. Pennycook, and D. G. Rand. Scaling up fact-checking using the wisdom of crowds. *Science Advances*, 7(36):eabf4393, 2021.

[4] R. DerSimonian and N. Laird. Meta-analysis in clinical trials. *Controlled Clinical Trials*, 7(3):177–188, 1986.

[5] N. Dias, G. Pennycook, and D. G. Rand. Emphasizing publishers does not effectively reduce susceptibility to misinformation on social media. *Harvard Kennedy School (HKS) Misinformation Review*, 1, 2020.

[6] D. Fudenberg and J. Tirole. *Game Theory.* MIT Press, Cambridge, MA, 1991.

[7] J. P. T. Higgins and S. G. Thompson. Quantifying heterogeneity in a meta-analysis. *Statistics in Medicine*, 21(11):1539–1558, 2002.

[8] C. Hilbe, M. A. Nowak, and K. Sigmund. Evolution of extortion in iterated prisoner's dilemma games. *Proc Natl Acad Sci U S A*, 110(17):6913–8, Apr 2013.

[9] G. Pennycook, Z. Epstein, M. Mosleh, A. A. Arechar, D. Eckles, and D. G. Rand. Shifting attention to accuracy can reduce misinformation online. *Nature*, 592(7855):590–595, 2021.

[10] G. Pennycook and D. G. Rand. Fighting misinformation on social media using crowd-sourced judgments of news source quality. *Proceedings of the National Academy of Sciences*, 116(7):2521–2526, 2019.

[11] W. H. Press and F. J. Dyson. Iterated prisoner's dilemma contains strategies that dominate any evolutionary opponent. *Proc Natl Acad Sci U S A*, 109(26):10409–13, Jun 2012.

[12] W. H. Sandholm. *Population games and evolutionary dynamics.* Economic learning and social evolution. MIT Press, Cambridge, Mass., 2011.

[13] A. J. Stewart and J. B. Plotkin. Extortion and cooperation in the prisoner's dilemma. *Proc Natl Acad Sci U S A*, 109(26):10134–5, Jun 2012.

[14] A. J. Stewart and J. B. Plotkin. From extortion to generosity, evolution in the iterated prisoner's dilemma. *Proc Natl Acad Sci U S A*, 110(38):15348–53, Sep 2013.

[15] A. Traulsen, M. A. Nowak, and J. M. Pacheco. Stochastic dynamics of invasion and fixation. *Phys Rev E Stat Nonlin Soft Matter Phys*, 74(1 Pt 1):011909, Jul 2006.

[16] S. Vosoughi, D. Roy, and S. Aral. The spread of true and false news online. *Science*, 359(6380):1146–1151, 2018.
